# Efficacy of Zemedy, a Mobile Digital Therapeutic for the Self-management of Irritable Bowel Syndrome: Crossover Randomized Controlled Trial

**DOI:** 10.2196/26152

**Published:** 2021-05-20

**Authors:** Melissa Hunt, Sofia Miguez, Benji Dukas, Obinna Onwude, Sarah White

**Affiliations:** 1 Department of Psychology University of Pennsylvania Philadelphia, PA United States; 2 Bold Health Limited, UK London United Kingdom; 3 Population Health Research Institute St George’s University of London London United Kingdom

**Keywords:** digital health, irritable bowel syndrome, cognitive behavioral therapy, CBT, efficacy, mHealth, self-management, IBS, randomized controlled trial, app

## Abstract

**Background:**

Patients with irritable bowel syndrome (IBS) experience abdominal pain, altered bowel habits, and defecation-related anxiety, which can result in reduced productivity and impaired health-related quality of life (HRQL). Cognitive behavioral therapy (CBT) has been shown to reduce symptoms of IBS and to improve HRQL, but access to qualified therapists is limited. Smartphone-based digital therapeutic interventions have potential to increase access to guided CBT at scale, but require careful study to assess their benefits and risks.

**Objective:**

The aim of this study was to test the efficacy of a novel app, Zemedy, as a mobile digital therapeutic that delivers a comprehensive CBT program to individuals with IBS.

**Methods:**

This was a crossover randomized controlled trial. Participants were recruited online and randomly allocated to either immediate treatment (n=62) or waitlist control (n=59) groups. The Zemedy app consists of 8 modules focusing on psychoeducation, relaxation training, exercise, the cognitive model of stress management, applying CBT to IBS symptoms, reducing avoidance through exposure therapy, behavioral experiments, and information about diet. Users interact with a chatbot that presents the information and encourages specific plans, homework, and exercises. The treatment was fully automated, with no therapist involvement or communication. At baseline and after 8 weeks, participants were asked to complete the battery of primary (Irritable Bowel Syndrome Quality of Life [IBS-QOL], Gastrointestinal Symptom Rating Scale [GSRS]) and secondary (Fear of Food Questionnaire [FFQ], Visceral Sensitivity Index [VSI], Gastrointestinal Cognition Questionnaire [GI-COG], Depression Anxiety Stress Scale [DASS], and Patient Health Questionnaire-9 [PHQ-9]) outcome measures. Waitlist controls were then offered the opportunity to crossover to treatment. All participants were assessed once more at 3 months posttreatment.

**Results:**

Both intention-to-treat and completer analyses at posttreatment revealed significant improvement for the immediate treatment group compared to the waitlist control group on both primary and secondary outcome measures. Gains were generally maintained at 3 months posttreatment. Scores on the GSRS, IBS-QoL, GI-COG, VSI, and FFQ all improved significantly more in the treatment group (*F*_1,79_=20.49, *P*<.001, Cohen *d*=1.01; *F*_1,79_=20.12, *P*<.001, *d*=1.25; *F*_1,79_=34.71, *P*<.001, *d*=1.47; *F*_1,79_=18.7, *P*<.001, *d*=1.07; and *F*_1,79_=12.13, *P*=.001, *d*=0.62, respectively). Depression improved significantly as measured by the PHQ-9 (*F*_1,79_=10.5, *P*=.002, *d*=1.07), and the DASS Depression (*F*_1,79_=6.03, *P*=.02, *d*=.83) and Stress (*F*_1,79_=4.47, *P*=.04, *d*=0.65) subscales in the completer analysis but not in the intention-to-treat analysis. The impact of treatment on HRQL was mediated by reductions in catastrophizing and visceral sensitivity.

**Conclusions:**

Despite its relatively benign physical profile, IBS can be an extraordinarily debilitating condition. Zemedy is an effective modality to deliver CBT for individuals with IBS, and could increase accessibility of this evidence-based treatment.

**Trial Registration:**

ClinicalTrials.gov NCT04170686; https://www.clinicaltrials.gov/ct2/show/NCT04170686

## Introduction

### Background

Irritable bowel syndrome (IBS) is a chronic gastrointestinal (GI) disorder of multifactorial etiology that is characterized by abnormal centralized pain processing. IBS is defined by recurrent abdominal pain occurring at least one day per week in the last 3 months, associated with two or more of the following: related to defecation, associated with changes in the frequency or form of bowel movements (ie, characterized by constipation, diarrhea, or an alternating mix of the two). IBS is highly prevalent, affecting up to 10% of the US population. Many studies have demonstrated that IBS has high rates of psychiatric comorbidity (up to 90% in treatment-seeking patients) [1,2], and causes social and occupational impairment [3]. Beyond the core symptoms of abdominal pain and altered bowel habits, individuals with IBS suffer from a host of related difficulties that substantially impair health-related quality of life (HRQL) and functioning. Visceral hypersensitivity, common among IBS patients, is a phenomenon in which people feel normal gut sensations that most people would be unaware of, and experience many of these sensations as more painful compared with healthy controls [4]. Anxiety and visceral hypersensitivity are highly correlated [5]. Anxiety and hypervigilance related to the sensations exacerbate the hypersensitivity [6].

Illness-related anxiety is high among patients with IBS, and is a better predictor of impairment in quality of life than actual symptom severity [7]. A major component of this anxiety is “catastrophizing,” in which individuals envision the worst possible outcome of their GI symptoms and in turn develop maladaptive coping strategies [3]. Catastrophizing is highly correlated with impairment in HQRL for patients with IBS [8]. Because of their catastrophizing, many individuals with IBS engage in significant avoidance behavior that can easily meet the diagnostic criteria for agoraphobia [9].

### Cognitive Behavioral Therapy for IBS

Over the past two decades, cognitive behavioral therapy (CBT) has repeatedly proven to be an efficacious treatment for individuals suffering from IBS [10,11]. Specifically, there is empirical support that CBT reduces GI symptom severity and impairment in quality of life [12,13]. These CBT treatments typically include components of psychoeducation about the brain-gut axis, mindfulness and relaxation training [14], reducing automatic negative thoughts related to GI catastrophizing [15], exposure therapy to feared and avoided sensations and situations [16], and reducing visceral hypersensitivity [12]. One meta-analysis including 20 psychological treatments for IBS found that GI cognition change and GI-specific anxiety were important mediators in improving GI-related quality of life and GI symptom severity [17].

Although CBT is a promising treatment, access to IBS-specific CBT remains low for patients. There is a lack of clinicians competent in delivering GI-specific CBT [3]. Additionally, the cost of treatment looms high; individuals often lack insurance coverage for psychotherapy and must pay out of pocket, which can be burdensome given the hundreds of dollars their IBS likely already costs them [18]. It is therefore necessary to develop a cheaper, more easily accessible alternative mode of treatment.

Many groups have tested variants of CBT for IBS with limited or distant therapist involvement (eg, via email) [15,19] and typically obtain robust effect sizes. These studies generally showed that web-based and telephone-based CBT improved IBS more than treatment as usual (eg, [20]). Several treatment manuals and self-help books are available that detail the CBT treatment approach, and one [21] was found to be efficacious as a stand-alone treatment in a randomized controlled trial (RCT) [22].

In today’s digitized world, the mobile health (mHealth) industry is growing. The industry is currently valued at close to US $50 billion and is expected to multiply by nearly five times over the next decade [23]. Thousands of mobile apps exist to improve health across the spectrum. Mobile apps have multiple advantages, including low cost, privacy, accessibility, and convenience for the user.

CBT is among the forms of treatment increasingly being delivered via apps. In their review of eight CBT apps, Rathbone et al [24] found that CBT self-help apps can be efficacious, most notably in alleviating depressive symptoms. They also cited the willingness of participants to engage in therapy as a key component of the apps’ success [24]. A component of many mHealth apps, and specifically those that use CBT, is automated guidance and feedback. Automated guidance has been found to be effective in reducing substance abuse among urban women and emerging adults [25,26]. Kelders et al [27] compared an automated treatment for depression with standard, in-person clinical treatment and found that depressive symptoms were moderately reduced for those in the automated group, although not as strongly as found for the in-person treatment group. However, Mason and Andrews’ [28] internet CBT study found that “specialist assessments and initial face-to-face contact do not influence treatment outcome, and that patients do just as well with an automated assessment.” Hauser-Ulrich et al [29] developed a smartphone app to treat chronic pain through CBT. This app employs a chatbot that guides users through modules [29]. In their RCT, the authors found improvements in pain-related impairment, pain intensity, and general well-being for those who used the app for 8 weeks [29]. Thus, there is strong evidence to suggest that automated treatment in a CBT app may be highly effective in delivering integrative behavioral health care for patients with disorders at the boundary between physical symptoms and psychological distress.

### Aim

As self-help modalities are increasingly available online and through smartphone apps, it is important to test the efficacy of those apps through rigorous, controlled research. The purpose of this study was to test the efficacy of a novel digital app (Zemedy) that applies CBT to IBS.

## Methods

### Trial Registration

This study was approved by the Institutional Review Board of the University of Pennsylvania. All participants provided electronic consent prior to participation in the study. The deidentified dataset analyzed in the study is available from the corresponding author upon request. This trial was registered at ClinicalTrials.gov as NCT04170686.

### App Description

**Figure 1:**
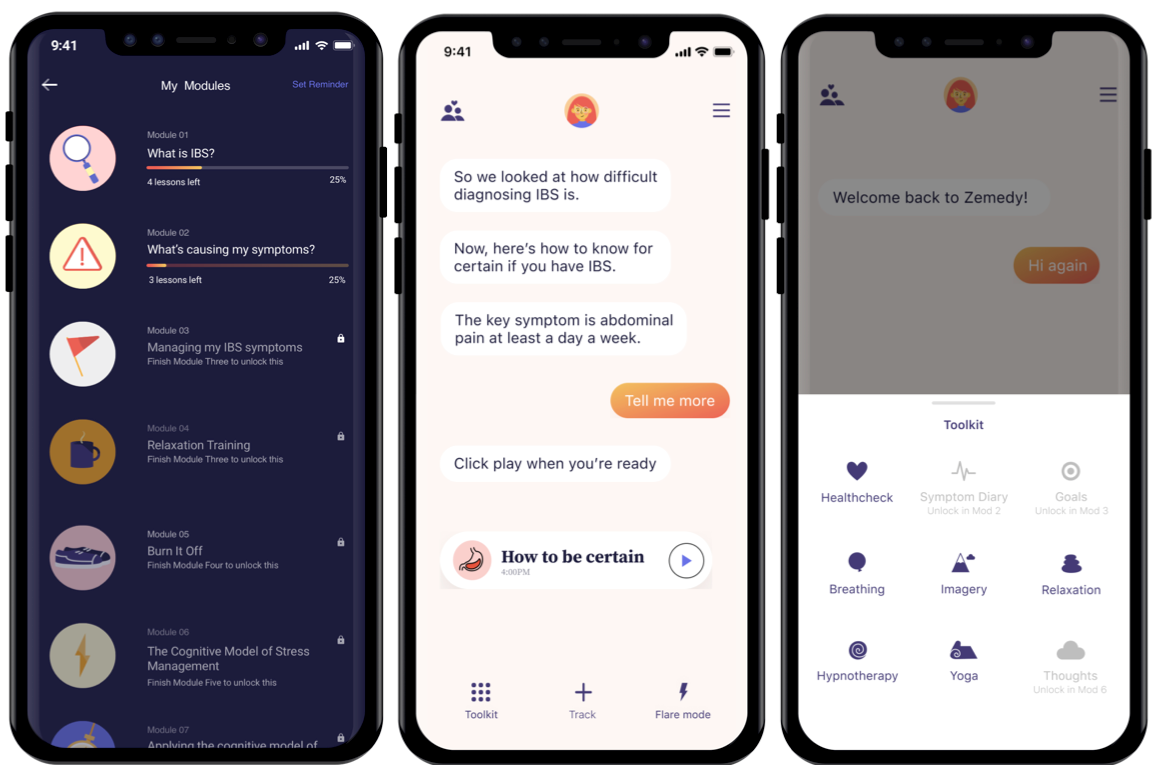
Screenshots of Zemedy.

Zemedy 1.0 is a mobile phone app designed by Bold Health, a UK-based digital health company, in collaboration with the principal investigator (MH) based on her empirically supported self-help book [21]. The app treats IBS through CBT specifically developed for the condition. Users of either iOS or Android smartphones are guided through the app by a chatbot with whom they “text.” The app consists of 10 modules. The first two modules are devoted to psychoeducation about the etiology of IBS and CBT’s effectiveness in treating it. The remaining eight modules teach users about various CBT strategies to mitigate the impact of IBS on daily life, including relaxation training, exercise, cognitive restructuring and decatastrophizing, exposure exercises to reduce avoidance, and behavioral experiments. It also encourages a healthy (but not highly restrictive) diet. See Figure 1 for screenshots of the Zemedy app. Users are prompted to apply these strategies to their daily lives. Similar to the chronic pain treatment developed by Hauser-Ulrich et al [29], Zemedy is designed to be completed in 8 weeks. Participants were encouraged to read through the first 5 modules (education, relaxation training, and exercise) in the first week, and to practice relaxation exercises daily. The remaining modules were designed to be worked through approximately one per week, with practice and homework exercises performed daily to learn and apply the skills.

The app also includes a “flare module,” which users can access at any point to address immediate GI pain and anxiety. Shah et al [14] found that mind-body interventions such as relaxation training and hypnosis have moderate effect sizes in reducing IBS symptoms. The flare module contains a variety of exercises such as deep breathing, progressive muscle relaxation, relaxation imagery, and hypnotherapy scripts that help mitigate distress and discomfort in the moment.

Participants were provided with a link to download the app. They were provided the app at no cost. The entire intervention was delivered within the app with no human involvement (eg, therapist guidance or feedback). If participants experienced technical difficulties, they could reach out to technical support at Bold Health. They received a single email at 4 weeks from a research coordinator in the trial providing general encouragement to continue working through the app (if they were in the immediate treatment group) or to “hang in there” (if they were in the waitlist control group).

### Design

This was a randomized waitlist control crossover trial with assessments performed at baseline, postintervention (8 weeks), following crossover to intervention for the waitlist control group, and at follow-up (3 months postintervention). After completing the consent and all baseline measures, participants were randomly allocated by a research coordinator to either the immediate treatment or waitlist control group using the coin toss function of random.org. After 8 weeks, all participants were asked to complete the same battery of measures. At that point, participants in the waitlist group were crossed over and were given access to the app. After 8 weeks of access, they were asked to complete the battery of questionnaires again. All participants were then assessed one final time 3 months after completing the treatment phase. Unfortunately, the onset of the COVID-19 pandemic coincided with the follow-up portion of the trial, and it is unclear the extent to which the pandemic affected both attrition and long-term results.

### Sample Size

The power analysis showed that 30 participants per randomized group would have 85% statistical power at a two-sided significance level of .05 to detect an effect size of 0.76. The effect size was chosen as previous studies of internet-delivered CBT for IBS reported similar effect sizes for HRQL and GI symptoms outcomes [15,22]. Assuming 50% attrition, as is common in internet-based intervention studies, we aimed to recruit 120 participants to have ample power to detect main effects, and to explore potential mediators and moderators. 

### Inclusion and Exclusion Criteria

Inclusion criteria consisted of being 18 years of age or older, and reporting having been previously diagnosed by a physician with IBS or meeting the Rome IV criteria by self-report. We did not specify a time frame for the physician diagnosis; therefore, it is possible that some participants were originally diagnosed under Rome III criteria. Owning a smartphone and computer/internet literacy were de facto eligibility criteria.

Exclusion criteria consisted of reporting having received a diagnosis of another comorbid GI disorder such as celiac disease or an inflammatory bowel disease. Exclusion criteria also included severe depression and/or suicidal ideation, defined as a score of 20 or above on the Patient Health Questionnaire-9 (PHQ-9), and/or positive endorsement of active suicidal ideation or intent on a separate suicide question. Twenty-five individuals met this criterion. They were excluded from the trial, but were given immediate access to the app. The principal investigator, who is a licensed clinical psychologist, followed up with each of these individuals to complete a risk assessment and offer other resources such as referrals to local in-person providers. 

### Participants

A total of 146 potential participants were screened, 121 of whom met the inclusion criteria. Participants were recruited for the trial through IBS-specific social media sites with a combination of graphic advertisements, and posts and comments on threads informing site users about the study. Most participants came to the study through Facebook (n=30), Twitter (n=32), and the IBS subReddit (n=51). There were no face-to-face components to the trial in terms of recruitment, assessment, or intervention. Posts and advertisements included a link to a secure University of Pennsylvania Qualtrics study page. On following the link, potential participants would first see the detailed explanation of the research (consent form; see Multimedia Appendix 1) and would consent to completing the baseline questionnaires. Questionnaires were completed via Qualtrics and could be downloaded securely by the research team. Participants were identified by email during data collection. All data were stored in a deidentified format. All recruitment and follow-up occurred between October 1, 2019 and November 1, 2020. The trial ended upon successful completion.

All but five participants reported that they had been diagnosed with IBS by a physician at some point (which could have been under Rome III or Rome IV criteria). The five participants who did not report a physician diagnosis all met stringent Rome IV criteria by self-report. Of the 30 (24.8%) participants who reported a physician diagnosis but did not meet stringent Rome IV criteria, 7 reported pain 3 days a month and would have met Rome III criteria. Another 5 participants reported even less frequent pain. Four women reported pain solely during their menstrual period. Four people failed to meet the duration criteria (less than 6 months total since onset). The final 10 participants met only one, rather than two, of the three criteria beyond frequent abdominal pain.

With respect to IBS subtype, 48 (39.7%) participants reported diarrhea-predominant IBS, 28 (23.1%) reported mixed subtype, 11 (9.1%) reported constipation-predominant IBS, and 4 (3.3%) reported undifferentiated IBS. The remaining 30 (who did not meet all Rome IV criteria) were not divided into subtypes.

### Randomization and Blinding

Participants who met the inclusion criteria were allocated to condition using the coin toss feature of random.org. A total of 62 participants were assigned to the immediate treatment condition and 59 were assigned to the waitlist control. The allocation sequence was concealed to participants until they were enrolled, had completed baseline data collection, and had been assigned to a group. The majority of baseline symptom severity measures were not significantly different between the immediate treatment and waitlist control groups. However, participants in the waitlist control group reported significantly more depression and more impaired HRQL than those in the immediate treatment group. Although the design should have yielded a low risk of bias from randomization, the slight differences in symptom severity at baseline suggest some concerns about randomization, according to the Cochrane risk of bias tool [30].

Because of the nature of the trial (immediate treatment versus waitlist control group), neither participants nor research coordinators were blinded to condition. However, there were no deviations from the intended intervention. Moreover, all outcome data were self-reported. Thus, blinding of evaluators was neither possible nor necessary.

### Procedure

Participants in the immediate treatment group were given the link to access the Zemedy app, and were encouraged to download it and begin working through the modules immediately. The waitlist control group was told they would be given access to the app in 8 weeks. Four weeks after baseline, participants in the treatment group were emailed to encourage them to continue using the app, and the waitlist control group was emailed to offer encouragement, remind them they were still enrolled in the study, and let them know that they would be receiving the follow-up questionnaires in 4 weeks.

Eight weeks after completing the baseline questionnaire, all participants were emailed a link to a second Qualtrics page that contained all of the same measures as completed at baseline. Those in the waitlist control condition were then given access to the app.

After having had access to the app for 8 weeks, participants in the waitlist control group were emailed a link to the third battery of questionnaires that was identical to the battery received by the treatment group after 8 weeks of app usage, which included the same measures as contained in the baseline battery.

Finally, all participants were emailed a final link to the last battery of questionnaires (again identical to the battery at baseline and posttreatment) 3 months after they completed the active treatment phase. Upon completion of each round of questionnaires, participants received US $20 in Amazon credit.

If at any point a participant had indicated a significant increase in depressive symptoms or the onset of suicidal ideation, the team would have alerted the principal investigator (a licensed clinical psychologist) who would have reached out to that individual to perform a risk assessment and offer referrals to local resources. No such adverse events occurred.

### Primary Outcome Measures

#### IBS-Quality of Life

The IBS-Quality of Life (IBS-QOL) questionnaire is a 34-item self-report measure specific to IBS designed to assess the impact of IBS on quality of life [31,32]. The IBS-QOL has high internal consistency (Cronbach α=.95), high reproducibility (intraclass correlation coefficient=0.86), and good construct validity [32]. Qualitative score ranges are 0-31 (minimal or mild), 32-66 (moderate), and 67-100 (severe impairment).

#### Gastrointestinal Symptom Rating Scale-IBS

The Gastrointestinal Symptom Rating Scale-IBS (GSRS-IBS) contains 13 self-report items rated on a 6-point Likert scale ranging from 1 (no discomfort at all) to 7 (very severe discomfort) [33]. Total scores range from 0 to 78. The GSRS-IBS has 5 subscales, including abdominal pain, bloating, constipation, diarrhea, and satiety. Each dimension has demonstrated high internal consistency, with Cronbach α ranging from .74 (pain) to .85 (satiety). Furthermore, the GSRS- IBS has demonstrated both high test-retest reliability, with intraclass correlations among the factors ranging from 0.55 (pain) to 0.70 (bloating), as well as high construct validity [33]. Overall internal consistency was good in our sample with Cronbach α=.81. The GSRS has been used as a primary outcome measure in several recent RCTs of IBS treatments [9], and the Rome Foundation reports that it is shorter and more user-friendly than the IBS Severity Scoring System [34]. Qualitative score ranges are 0-20 (minimal or mild), 21-39 (moderate), and 40-78 (severe).

### Secondary Outcome Measures

#### Rome IV Criteria Questionnaire

We used a questionnaire to determine whether participants met the current Rome IV diagnostic criteria for IBS. Our questionnaire was based on the Rome IV IBS-Specific Questionnaire, which is a validated self-report scale that covers the diagnostic criteria for IBS. It has been found to have acceptable sensitivity and high specificity as well as good test-retest reliability [35]. We used a modified, shortened version with 10 items that covered all diagnostic criteria.

#### Fear of Food Questionnaire

The Fear of Food Questionnaire (FFQ) is an 18-item self-report questionnaire that measures fear, avoidance of food, as well as life interference and loss of pleasure from eating [36]. Items are rated on a Likert scale ranging from 0 (not at all) to 5 (absolutely). It has excellent internal consistency reliability with Cronbach α=.96 and strong 2-week test-retest reliability at r=0.93, *P*<.001 [36]. The FFQ also shows good criterion and known-groups validity [36]. Qualitative score ranges are 0-15 (minimal), 16-30 (mild), 31-45 (moderate), and 46-90 (severe).

#### Visceral Sensitivity Index

The Visceral Sensitivity Index (VSI) is a unidimensional 15-item scale that measures GI symptom–specific anxiety [6,37]. Items are rated on a Likert scale ranging from 0 (strongly disagree) to 5 (strongly agree). It has high internal consistency (Cronbach α=.93) and a mean interitem correlation of 0.47 [37,38]. It has good criterion, construct, and predictive validity [6]. Qualitative score ranges are 0-10 (minimal or mild), 11-30 (moderate), and 31-75 (severe).

#### Gastrointestinal Cognitions Questionnaire

The Gastrointestinal Cognitions Questionnaire (GI-COG) consists of 16 self-report items that are rated on a 5-point Likert scale, ranging from 0 (hardly) to 4 (very much). Individual items are summed and total scores range from 0 to 64. The questionnaire consists of three subscales: the pain/life interference subscale (eg, “When I feel my GI symptoms acting up, I’m afraid the pain will be excruciating and intolerable”), the social anxiety subscale (eg, “If I have to get up and leave an event, meeting, or social gathering to go to the bathroom people will think there’s something wrong with me”), and the disgust sensitivity subscale (eg, “The thought of fecal incontinence is terrifying. If it happened, it would be awful”). The GI-COG has been shown to have excellent internal consistency (Cronbach α=.92) and test-retest reliability (r=0.87, *P*=.001) [39]. Qualitative score ranges are 0-19 (minimal or mild), 20-39 (moderate), and 40-64 (severe).

#### Depression Anxiety Stress Scale

The Depression Anxiety Stress Scale (DASS) is a 42-item self-administered questionnaire that measures the magnitude of depression, anxiety, and stress independently. Internal consistency for each of the subscales of the questionnaire are high, with Cronbach α of .96 to .97 for DASS-Depression, .84 to .92 for DASS-Anxiety, and .90 to .95 for DASS-Stress [40,41]. The DASS has been found to be a highly reliable and valid measure of the constructs it is intended to assess [42].

#### PHQ-9 Assessment

The PHQ-9 is a depression scale that consists of 9 self-report items. The 9 items aim to quantify the 9 criteria upon which the diagnosis of depressive disorders is based in the Diagnostic and Statistical Manual of Mental Disorders-IV. The PHQ-9 can establish a depressive disorder diagnosis and depressive symptom severity [43]. Each of the 9 items can be scored from 0 (not at all) to 3 (nearly every day); therefore, scores can range from 0 to 27. The PHQ-9 has been found to demonstrate high internal reliability, with Cronbach α of .89 when tested in a primary care setting and .86 when tested in an obstetrics-gynecology setting [43].

#### Dose

Dosage was measured according to the number of modules completed. The mobile app sent usage data to the backend system each time a participant visited the app. Data include the time and date of each session on the app.

### Statistical Analysis

Univariate general linear models in SPSS V25 were used to examine between-group effects at posttreatment (8 weeks), controlling for baseline levels of the dependent variable. Paired-sample *t* tests were used to examine within-group changes over the treatment phase for each group and maintenance of gains from posttreatment to 3-month follow-up. The robustness of these analyses was examined in an intention-to-treat sensitivity analysis using multiple imputation. As shown below, missing data at follow-up were not entirely missing at random. Therefore, baseline outcome measures were included in the imputation model as predictors together with the follow-up set of measures with missing data and imputation using the fully conditional specification [44] performed to create 15 imputed datasets. Regression models were then fitted as in the primary analysis, and pooled estimates of the treatment effect were calculated. Three sets of imputed datasets were created, one for each follow-up data point, with baseline measures included in each.

Change in visceral anxiety, catastrophizing, and fear of food (calculated as the change from baseline to 8 weeks) were explored as possible mediators of GI symptoms and quality of life at 8 weeks using regression analysis with estimates of indirect effects calculated using a percentile bootstrap estimation approach with 5000 samples implemented with the PROCESS macro Version 3.5 [45]. Both direct and indirect effects are reported. The direct effect quantifies the estimated difference in the dependent variable (GI symptoms or quality of life) between two cases that are equal on the mediator but differ by one unit on treatment assignment (ie, intervention vs waitlist group). The indirect effect quantifies how much two cases, one assigned to immediate treatment and the other to waitlist, are estimated to differ on the dependent variables (GI symptoms or quality of life) as a result of the treatment’s influence on the mediator, which in turn influences the dependent variable. Two sets of models were fitted: the first tested the mediator variables separately with simple mediator models, and the second fitted a parallel mediator model where the three mediators were tested simultaneously. The baseline level of the dependent variable was included as a covariate in all mediation models.

## Results

### Participant Characteristics

**Figure 2:**
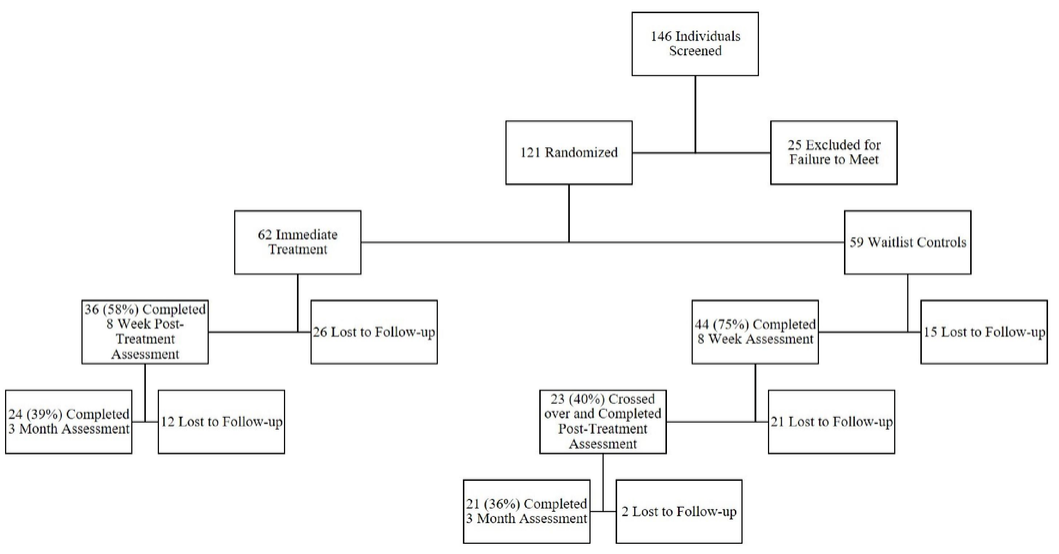
CONSORT diagram of participant flow through the study.

The mean participant age was 32 years (SD 10.2, range 18-63). Of the total 121 participants, 76.0% (n=92) were white, 5.8% (n=7) were Hispanic, 5.0% (n=6) were black, 4.1% (n=5) were Asian, and the remaining 9.1% (n=11) identified as mixed race or other. With respect to gender, 75.2% (91/121) identified as female and 24.8% (30/121) identified as male. With respect to marital status, 43.0% (52/121) reported being single, 32.2% (39/121) reported being married, 19.0% (23/121) reported having a partner or cohabiting, and 5.8% (7/121) reported being divorced at baseline. With respect to employment, 22.3% (27/121) of the participants were students, 15.7% (19/121) reported being employed part time, 47.9% (58/121) reported being employed full time, and 14.0% (17/121) reported that they were not working when completing the baseline surveys. See Figure 2 for the CONSORT diagram of participant flow through the study.

There were no significant differences between the immediate treatment and waitlist control groups on any of the demographic variables, or in baseline GI symptoms, visceral sensitivity, catastrophizing, or fear of food. However, as noted above, the waitlist control group was found to be slightly more distressed than the treatment group at baseline. The waitlist control group reported significantly more depression (PHQ-9, t_119_=2.99, *P*=.003; DASS-Depression t_119_=2.11, *P*=.04) and more impaired quality of life (t_119_=2.04, all *P*=.04) than the immediate treatment group, although effect sizes were modest (*d*=0.38 for the DASS, *d*=0.54 for PHQ-9, and *d*=0.37 for IBS-QOL). Thus, baseline symptoms were controlled in all analyses.

There were no univariate outliers found at baseline.

### Outcomes

Completer analyses assessing the impact of treatment on outcome at 8 weeks revealed significant improvement for the immediate treatment group, relative to the waitlist control group, for both primary outcomes of GI symptom severity and HRQL (*F*_1,79_=20.12, *P*<.001, Cohen *d*=1.02 and *F*_1,79_=20.49, *P*<.001, *d*=1.25, respectively). With respect to the secondary outcome measures, GI-specific catastrophizing, visceral anxiety, and fear of food all improved significantly more in the treatment group (*F*_1,79_=34.71, *P*<.001, *d*=1.47; *F*_1,79_=18.7, *P*<.001, *d*=1.07; and *F*_1,79_=12.13, *P*=.001, *d*=0.62, respectively). Finally, depression improved significantly more in the immediate treatment group as measured by both the PHQ-9 (*F*_1,79_=10.5, *P*=.002, *d*=1.07), and the DASS Depression (*F*_1,79_=6.03, *P*=.02, *d*=0.83) and Stress (*F*_1,79_=4.47, *P*=.04, *d*=0.65) subscales. Only the DASS Anxiety subscale failed to show a significant advantage for the treatment group (*F*_1,79_=1.84, *P*=.18, *d*=0.41). See Table 1 for all means and SDs across all assessment timepoints. These results were replicated in the intention-to-treat analyses using multiple imputation, with the exception of the PHQ-9 and DASS scores, which were nonsignificant (Table 2).

For the immediate treatment group, all of the outcome variables changed significantly from pretreatment to posttreatment with the exception of the DASS Depression subscale, which showed only marginally significant improvement (Table 3). Sensitivity analysis using multiple imputation found the same pattern of significance.

**Table 1:** Mean (SD) outcome measures across the trial for the immediate treatment and waitlist control groups.

Outcome		Baseline			Eight weeks			Waitlist posttreatment (n=23)		Three months	
		Immediate treatment (n=62)	Waitlist control (n=59)	Immediate treatment (n=36)		Waitlist control (n=44)			Immediate treatment (n=24)		Waitlist control (n=21)
IBS-QOL^a^		53.63 (18.67)	60.48 (18.29)	34.25 (19.78)		58.19 (18.53)	76.6 (20.07)		38.08 (18.42)		43.98 (21.1)
GSRS^b^		36.76 (12.77)	37.75 (12.02)	27.56 (10.12)		38.18 (10.79)	34.26 (14.98)		27.83 (9.37)		30.95 (11.88)
GI-COG^c^		36.92 (13.35)	40.07 (12.04)	22.44 (13.72)		40.84 (11.23)	33.3 (12.34)		23.75 (12.06)		31.71 (14.11)
VSI^d^		51.74 (12.29)	53.54 (11.44)	38.14 (16.21)		53.57 (12.37)	46.43 (12.78)		41.08 (14.13)		45.00 (12.63)
FFQ^e^		52.87 (19.14)	55.46 (18.21)	41.22 (22.23)		53.75 (18.08)	46.10 (19.87)		42.83 (20.99)		42.38 (19.87)
PHQ^f^		8.32 (5.29)	11.03 (4.66)	5.78 (4.20)		10.32 (4.29)	10.30 (5.80)		6.92 (5.71)		10.33 (5.97)
**DASS^g^**											
	Depression	11.65 (9.88)	15.59 (10.69)	7.83 (7.88)		15.45 (10.39)	14.43 (10.89)		9.08 (8.26)		16.38 (12.89)
	Stress	17.84 (9.56)	18.71 (8.97)	12.72 (8.65)		18.82 (9.99)	18.78 (10.03)		15.08 (8.40)		16.86 (9.69)
	Anxiety	12.03 (7.35)	12.19 (9.14)	8.67 (6.38)		12.05 (9.72)	11.83 (9.72)		9.08 (7.76)		10.00 (6.99)

^a^IBS-QOL: IBS Quality of Life.

^b^GSRS: Gastrointestinal Symptom Rating Scale.

^c^GI-COG: Gastrointestinal Cognitions Questionnaire.

^d^VSI: Visceral Sensitivity Index.

^e^FFQ: Fear of Food Questionnaire.

^f^PHQ: Patient Health Questionnaire.

^g^DASS: Depression Anxiety Stress Scale.

**Table 2:** Significance of treatment allocation at 8 weeks using multiple imputation.

Measure		*t* value	*P* value (two-tailed)
IBS-QOL^a^		–2.8	.005
GSRS^b^		–2.8	.005
GI-COG^c^		–3.4	.001
VSI^d^		–2.8	.006
FFQ^e^		–2.4	.02
PHQ^f^		–1.7	.08
**DASS^g^**			
	Depression	–1.3	.21
	Stress	–1.2	.25
	Anxiety	–0.6	.55

^a^IBS-QOL: IBS Quality of Life.

^b^GSRS: Gastrointestinal Symptom Rating Scale.

^c^GI-COG: Gastrointestinal Cognitions Questionnaire.

^d^VSI: Visceral Sensitivity Index.

^e^FFQ: Fear of Food Questionnaire.

^f^PHQ: Patient Health Questionnaire.

^g^DASS: Depression Anxiety Stress Scale.

**Table 3:** Improvement from baseline to posttreatment for the immediate treatment group.

Measure		*t* value (*df*=35)	*P* value (two-tailed)
IBS-QOL^a^		4.368	<.001
GSRS^b^		3.312	.002
GI-COG^c^		5.603	<.001
VSI^d^		3.454	.001
FFQ^e^		3.523	.001
PHQ^f^		2.327	.03
**DASS^g^**			
	Depression	1.707	.10
	Stress	2.273	.03
	Anxiety	2.164	.04

^a^IBS-QOL: IBS Quality of Life.

^b^GSRS: Gastrointestinal Symptom Rating Scale.

^c^GI-COG: Gastrointestinal Cognitions Questionnaire.

^d^VSI: Visceral Sensitivity Index.

^e^FFQ: Fear of Food Questionnaire.

^f^PHQ: Patient Health Questionnaire.

^g^DASS: Depression Anxiety Stress Scale.

### Clinically Significant Change

In terms of *clinically* significant change, we used Criterion B (falling within 2 SD of the healthy mean), which is more conservative than Criterion A (falling 2 SD below the pathological mean) [46]. For GI symptoms, the mean GSRS score for healthy controls is 12 (SD 11), leading to a cut-off point of 34. In the immediate treatment group, 24 out of 36 participants (66%) met this criterion at posttreatment. For HRQL, the mean IBS-QOL score for healthy controls is 5 (SD 11), leading to a cut-off point of 27. In the immediate treatment group, 16 out of 36 participants (44%) met this criterion at posttreatment. From another perspective, the qualitative range for minimal to mild impairment on the IBS-QOL is 0-31. An additional 2 participants would meet this slightly less stringent criterion, leading to a total of 50% of participants in the immediate treatment group showing an excellent response. This yields a number needed to treat of 2.

After completing the 8-week follow-up questionnaires, the waitlist group was crossed over to active treatment and was given access to the app for 8 weeks. Paired-samples *t* tests comparing their scores at the initial 8-week follow-up to their scores posttreatment revealed significant improvement in HRQL, catastrophizing, visceral anxiety, and fear of food, but not on GI symptoms, depression, or anxiety (Table 4). Sensitivity analysis using multiple imputation showed a similar pattern of significance at the 5% level but with lower *P* values closer to the significance level.

**Table 4:** Improvement in the waitlist control group after crossover to active treatment.

Measure		*t* value (*df*=22)	*P* value (two-tailed)
IBS-QOL^a^		–3.124	.005
GSRS^b^		–1.308	.20
GI-COG^c^		–2.748	.01
VSI^d^		–2.618	.02
FFQ^e^		–3.509	.002
PHQ^f^		0.103	.92
**DASS^g^**			
	Depression	–1.537	.14
	Stress	–0.361	.72
	Anxiety	0.360	.72

^a^IBS-QOL: IBS Quality of Life.

^b^GSRS: Gastrointestinal Symptom Rating Scale.

^c^GI-COG: Gastrointestinal Cognitions Questionnaire.

^d^VSI: Visceral Sensitivity Index.

^e^FFQ: Fear of Food Questionnaire.

^f^PHQ: Patient Health Questionnaire.

^g^DASS: Depression Anxiety Stress Scale.

Three-month follow-up data were collected for all participants (both the immediate treatment group and the waitlist group who had been crossed over to treatment) between March and October of 2020. Unfortunately, this meant that all follow-up data were collected after the onset of the COVID-19 pandemic. Nevertheless, participants (all of whom had had access to the active treatment at this point) continued to show significant improvement over baseline on all outcome variables except depression (Table 5).

**Table 5:** Difference between baseline and 3-month follow-up data for all participants (N=121).

Measure		*t* value *(df*=44*)*	*P* value (two-tailed)
IBS-QOL^a^		5.136	<.001
GSRS^b^		4.064	<.001
GI-COG^c^		6.090	<.001
VSI^d^		4.261	<.001
FFQ^e^		4.000	<.001
PHQ^f^		1.489	.14
**DASS^g^**			
	Depression	0.499	.62
	Stress	2.264	.03
	Anxiety	3.012	.004

^a^IBS-QOL: IBS Quality of Life.

^b^GSRS: Gastrointestinal Symptom Rating Scale.

^c^GI-COG: Gastrointestinal Cognitions Questionnaire.

^d^VSI: Visceral Sensitivity Index.

^e^FFQ: Fear of Food Questionnaire.

^f^PHQ: Patient Health Questionnaire.

^g^DASS: Depression Anxiety Stress Scale.

Finally, we assessed maintenance of treatment gains from posttreatment to 3-month follow-up. Without exception, gains were maintained, and there were no significant changes or relapse in symptoms, except for a slight rise in depression. Thus, even in the face of an incredibly stressful global pandemic, by and large, our participants showed remarkable resilience, and their HRQL, GI symptoms, GI-specific catastrophizing, anxiety, and fear of food remained much improved (Table 6). This result was confirmed in a sensitivity analysis using multiple imputation (Table 7).

**Table 6:** Maintenance of gains from posttreatment to 3 months.

Measure		*t* value (*df*=43)	*P* value (two-tailed)
IBS-QOL^a^		0.289	.77
GSRS^b^		0.636	.53
GI-COG^c^		0.841	.41
VSI^d^		0.056	.96
FFQ^e^		0.240	.81
PHQ^f^		–0.530	.60
**DASS^g^**			
	Depression	–1.614	.11
	Stress	0.335	.74
	Anxiety	0.935	.36

^a^IBS-QOL: Irritable Bowel Syndrome-Quality of Life.

^b^GSRS: Gastrointestinal Symptom Rating Scale.

^c^GI-COG: Gastrointestinal Cognitions Questionnaire.

^d^VSI: Visceral Sensitivity Index.

^e^FFQ: Fear of Food Questionnaire.

^f^PHQ: Patient Health Questionnaire.

^g^DASS: Depression Anxiety Stress Scale.

**Table 7:** Intention-to-treat sensitivity analysis of within-group changes using multiple imputation.

Measure		0-8 weeks, immediate treatment group (n=62)			8 weeks to posttreatment, waitlist group (n=59)			Posttreatment to 3-month follow-up, all participants (N=121)	
		*t* value	*P* value (two-tailed)	*t* value		*P* value (two-tailed)	*t* value		*P* value (two-tailed)
IBS-QOL^a^		4.7	<.001	3.5		.001	–0.47		.65
GSRS^b^		3.3	.001	2.0		.06	0.19		.85
GI-COG^c^		5.2	<.001	2.9		.008	0.10		.93
VSI^d^		3.7	<.001	2.4		.02	–0.26		.80
FFQ^e^		3.2	.002	2.0		.047	–0.15		.88
PHQ^f^		2.1	.04	2.0		.053	–0.32		.75
**DASS^g^**									
	Depression	1.2	.23	1.8		.08	–0.87		.39
	Stress	2.4	.02	1.5		.15	0.47		.64
	Anxiety	2.3	.02	1.9		.07	–0.14		.89

^a^IBS-QOL: Irritable Bowel Syndrome-Quality of Life.

^b^GSRS: Gastrointestinal Symptom Rating Scale.

^c^GI-COG: Gastrointestinal Cognitions Questionnaire.

^d^VSI: Visceral Sensitivity Index.

^e^FFQ: Fear of Food Questionnaire.

^f^PHQ: Patient Health Questionnaire.

^g^DASS: Depression Anxiety Stress Scale.

### Attrition

There was significant attrition from the study in both the immediate treatment and waitlist control groups (see Figure 2 for the flow chart of study enrollment). An independent-samples *t* test demonstrated that the only predictors of attrition at the 8-week follow-up were more severe visceral sensitivity (t_119_=2.18, *P*=.03) and fear of food (t_119_=1.79, *P*=.08) for participants in both the immediate treatment and waitlist group. About half of the participants (21 out of 44) in the waitlist control group who were offered crossover to active treatment were lost to follow-up at their posttreatment assessment. None of the measures at 8 weeks predicted attrition in this group. Of the 58 participants across both groups who completed the active treatment and the posttreatment questionnaires, 14 were lost to follow-up prior to the 3-month assessment. Participants who were lost to follow-up at that point were more likely to be *less* stressed (t_56_=2.19, *P*=.03), catastrophized *less* (t_56_=2.21, *P*=.03), and were somewhat *less* depressed (t_56_=1.72, *P*=.09) at posttreatment.

### Mediation

Another aim of the study was to test whether changes in catastrophic thinking, visceral sensitivity, and fear of food would at least partially mediate reductions in GI symptom severity and improvement in quality of life.

The simple mediator models for GI symptom severity showed that changes in visceral anxiety, catastrophizing, and fear of food were all significant mediators of the relationship between treatment and GI symptom severity. Participants assigned to immediate treatment had a greater decrease in visceral anxiety, catastrophizing, and fear of food, and participants who had a greater decrease in visceral anxiety, catastrophizing, and fear of food had lower GI symptom severity at 8 weeks while controlling for baseline GI symptom severity (Table 8). The statistically significant direct effect for each of the simple models indicated that treatment directly influenced GI symptom severity independent of the indirect effect of the mediating variables. The parallel multiple mediator model indicated that the indirect effects of visceral anxiety and fear of food were independent mediators, but the effect of catastrophizing was not significant (bias-corrected 95% CI included zero) and its effect is taken up by the other mediators. Once again there was a significant direct effect of treatment independent of mediators on GI symptom severity (*P*<.001).

Participants assigned to immediate treatment had a greater decrease in visceral anxiety, catastrophizing, and fear of food, and participants who had a greater decrease in visceral anxiety, catastrophizing, and fear of food had lower scores on IBS-QOL at 8 weeks while controlling for baseline IBS-QOL (Table 8). The statistically significant direct effect for the model including fear of food indicated that treatment directly influenced quality of life independent of the indirect effect of fear of food. However, having accounted for the effect of change in visceral anxiety and catastrophizing, no statistically significant direct effect of treatment remained. The parallel multiple mediator model indicated statistically significant indirect effects of the three mediators with no direct effect of treatment (Table 8).

**Table 8:** Direct and indirect mediation results.

Measure			GI^a^ symptom severity					IBS^b^ quality of life		
			Effect (95% BCI^c^)		*P* value		Effect (95% BCI)			*P* value
**Visceral anxiety**										
	Indirect	–4.3 (–7.0 to –1.8)		.002		–12.2 (–18.62 to –6.4)			<.001	
	Direct	–5.1 (–8.8 to –1.4)		.007		–4.6 (–9.8 to –.56)			.08	
**GI-specific catastrophizing**										
	Indirect	–3.7 (–7.1 to –1.2)		.007		–15.4 (–21.6 to –9.6)			<.001	
	Direct	–5.6 (–10.2 to –1.1)		.02		–1.4 (–7.9 to 5.1)			.67	
**Fear of food**										
	Indirect	–4.0 (–7.2 to –1.5)		.003		–9.8 (–16.3 to –3.8)			<.001	
	Direct	–5.4 (–9.1 to –1.6)		.005		–7.0 (–12.7 to –1.4)			.02	
**Parallel multiple mediator model**										
	Direct	–9.4 (–13.5 to –5.3)		<.001		–.5 (–5.2 to 4.2)			.83	
	Visceral anxiety	–3.4 (–6.2 to –1.0)		N/A^d^		–7.0 (–11.3 to –3.4)			N/A	
	COG^e^	1.8 (–1.0 to 4.4)		N/A		–5.1 (–9.0 to –1.8)			N/A	
	Fear of food	–2.7 (–5.5 to –0.8)		N/A		–4.3 (–8.3 to –1.2)			N/A	

^a^GI: gastrointestinal.

^b^IBS: irritable bowel syndrome.

^c^BCI: bias-corrected confidence interval.

^d^N/A: not applicable.

^e^COG: cognition.

### Moderation

Univariate analysis of the data revealed that Rome IV criteria moderated the effectiveness of the treatment. That is, there was a significant interaction between condition and Rome IV status such that the app was more helpful to the participants who reported meeting stringent Rome IV criteria for IBS at baseline than for those who did not, for both GI symptoms (*F*_3,76_=2.919, **P*=.04<.*0) and HRQL (*F*_3,76_=6.652, *P*=.001). The only difference at baseline between those who met the criteria and those who did not was severity of GI symptoms (t_144_=3.75, *P*<.001). No other baseline variables were significantly different. When the sample was restricted to *only* those individuals who met strict Rome IV criteria, the advantage of the treatment group over the waitlist group was even more marked for improvement in GI symptoms (*F*_1,56_=30.2, *P*<.001), HRQL (*F*_1,56_=47.42, *P*<.001), catastrophizing (*F*_1,56_=51.10, *P*<.001), visceral anxiety (*F*_1,56_=28.84, *P*<.001), and fear of food (*F*_1,56_=22.11, *P*<.001).

We also examined whether IBS subtype moderated the efficacy of the app; it did not.

### Dose-Dependent Response

Because the app itself tracks objective progress through the modules, we were able to examine the effect of “dose” (measured as components of the app accessed) on outcome. The majority of participants in the immediate treatment group who completed follow-up surveys finished just shy over 3 modules (mean 3.2, SD 2, median 2.9). Only one participant completed all possible modules. Dosage was marginally correlated with improvement in HRQL (*r*=0.33, *P*=.07) and depression (*r*=0.33, *P*=.08), but was not directly correlated with improvement in GI symptoms, changes in catastrophizing, or visceral anxiety. This suggests that the more participants used the app, the more their quality of life and depressive symptoms improved. These results also suggest that the primary change components in the app with respect to catastrophizing and visceral anxiety occurred early in the modules.

## Discussion

### Principal Findings

The purpose of this study was two-fold. First, we tested the efficacy of a cognitive behavioral intervention for IBS delivered via a digital self-help app, with no therapist feedback or involvement. Completer analyses yielded statistically and clinically significant improvement, with treatment having a positive impact on both GI symptom severity and quality of life. Intention-to-treat sensitivity analysis using multiple imputation replicated those findings. After treatment, individuals reported significantly lower levels of IBS symptoms and less impairment to their quality of life. Effect sizes for the primary outcomes and most of the secondary outcomes were all in the very large range. This 8-week intervention appears to have substantially reduced the burden of illness compared to that of waitlist controls.

Second, we tested whether reductions in IBS-specific catastrophic thinking, visceral sensitivity, and fear of food might mediate the efficacy of treatment. Reductions in these three variables did appear to mediate the impact of treatment on HRQL, but not on GI symptoms themselves. The app worked by reducing catastrophic thinking, visceral sensitivity to GI symptoms, and fear of food, which in turn improved individuals’ quality of life. This is consistent with prior findings about the impact of CBT on IBS. Additionally, changes in catastrophizing and visceral anxiety were observed in participants who had only completed the preliminary modules of the app. This is consistent with the idea that psychoeducation and relaxation can promote cognitive reframing and can reduce anxiety about visceral sensations.

Overall, we are strongly encouraged by the results of this study, which appear to suggest that effective CBT for IBS can be successfully delivered via an app. The Zemedy app seems to be an effective means to improve the lives of individuals with IBS. Zemedy, which is already in the App Store and Google Play Store for download, could dramatically increase the accessibility of effective treatment for this debilitating disorder.

### Limitations

This study had a number of limitations. The first was the lack of a placebo control condition. Patients with IBS typically show high placebo response rates [47], although the placebo effect is reduced when individuals meet more stringent (ie, Rome IV) diagnostic criteria. Future trials of the Zemedy app (including an ongoing trial registered as NCT04665271 on Clinicaltrials.gov) will include an active placebo control (sham app) rather than relying on a simple waitlist.

The second major limitation was the lack of rigorous diagnostic interviewing or explicit physician confirmation of the IBS diagnosis. Our inclusion criteria were self-reported as a prior physician diagnosis of IBS and/or meeting stringent Rome IV criteria. Five participants had no physician diagnosis but met Rome IV criteria. Thirty participants reported having been diagnosed by a physician but did not meet stringent Rome IV criteria. Of those, 7 would have met Rome III [1] criteria. The remaining individuals failed to meet either the duration or severity criteria.

The choice to include participants who did not meet Rome IV criteria was made because the aim of the study was to determine the efficacy of the app for individuals *who believe they have IBS and are searching for self-help materials*. The app will be accessible to all, and even those who perceive they have IBS without a clinical diagnosis or meeting criteria will use it. Thus, it is important to test the app among anyone who believes it to be relevant to their life. Interestingly, individuals who *did* meet criteria for IBS actually showed a significantly better response to the app. Thus, including individuals who might not have met strict Rome IV criteria is actually more conservative and more ecologically valid. The app includes educational material about the importance of a thorough differential diagnostic evaluation, and especially the importance of ruling out other potential causes of GI symptoms (such as celiac disease and inflammatory bowel diseases). Moving forward, it may be important for the app to encourage people who do *not* meet Rome IV criteria to consult with their physicians about other possible causes of their symptoms.

The third limitation was the rate of attrition, with 36% not completing follow-up measures. Of those who completed 8-week follow-up measures, most had not made it through a substantial portion of the app’s content. Nevertheless, the attrition rate from treatment of 36% is actually lower than the rate of 47% on average typically found in studies of online behavioral health interventions [48].

In addition, people did not drop out entirely at random. Participants who dropped out during the initial treatment phase had significantly higher rates of visceral anxiety and fear of food at baseline (although there were no other significant differences). Since CBT for IBS typically encourages acceptance of visceral sensations and reduction of behavioral avoidance (especially avoidance of food and food-related social situations), the treatment may have seemed particularly challenging for those individuals. This might represent a population requiring more personal guidance, encouragement, and support from in-person therapy.

A fourth limitation of the study was the inability to statistically establish the temporal precedence of the proposed mediators of change. In the study design, there was no midpoint survey to show that visceral anxiety, catastrophizing, and fear of food changed *before* quality of life improved. We did not include this intermediate survey during the treatment phase because we were concerned that it would increase attrition of participants, although a future study of the app would benefit from data obtained at this point.

A fifth limitation is that the PHQ-9 is a poor measure of depression severity because it only measures symptom frequency and does not take intensity of symptoms into account. For example, at baseline, someone might indicate feeling tired or having little energy nearly every day (scoring a 3), because they are so anergic they can barely get out of bed. By the end of a trial, they might still indicate feeling tired or having little energy nearly every day (scoring a 3) because they still feel chronically fatigued, but they are getting up and going to work every day. The *severity* of their anergia would have declined significantly, but the PHQ-9 would reflect no change. Furthermore, the item that assesses suicidality makes no distinctions at all with respect to passive versus active ideation, nor does it capture intent. An individual who has passive suicidal ideation daily, but no intent, would actually score *higher* than an individual who experiences less frequent, but intense active suicidal ideation with wavering intent. Although the PHQ-9 has been used in many other clinical trials of behavioral health interventions, and it *did* show significant improvement over the course of this trial in the completer sample (but not in the intention-to-treat analyses), we were dissatisfied with its sensitivity to treatment effects. Future studies of the app will employ more sensitive measures.

A sixth limitation is that we did not assess concurrent medication use. However, there is no reason to believe that medication use would have been different across the immediate treatment and control groups.

Finally, the last phase of the trial occurred during the COVID-19 global pandemic. Since all waitlist participants had already been crossed over to the active treatment phase, the 3-month follow-up data may be less reflective of the enduring effects of the treatment and more reflective of the massive social, economic, and personal upheaval the pandemic has caused. Indeed, the end of the treatment phase for all participants coincided with the COVID-19 pandemic’s arrival in the United States. With massive shutdowns and quarantines, it is highly likely that distress increased for all participants. The fact that treatment gains were generally maintained and that participants remained much improved over baseline (except for some recurrence of depression), even in the face of an unprecedented global health crisis, is encouraging.

### Conclusion

Despite the limitations, we believe that this study is of significant value. It successfully demonstrated the efficacy of an app that provided CBT for IBS patients. The intervention was not restricted by geography or scheduling constraints, and required no face-to-face contact with a clinician, aspects that dramatically increase the accessibility and portability of treatment. Despite its relatively benign physical profile, IBS can be an extraordinarily debilitating condition. Finding novel ways to disseminate evidence-based, effective treatments remains an important challenge, and Zemedy is a promising and effective way to help those suffering from IBS.

## Appendix

Multimedia Appendix 1 Consent form.

Multimedia Appendix 2 CONSORT-eHEALTH checklist (v 1.6.1).

## Abbreviations

CBT: cognitive behavioral therapy
DASS: Depression Anxiety Stress Scale
FFQ: Fear of Food Questionnaire
GI: gastrointestinal
GI-COG: Gastrointestinal Cognitions Questionnaire
GSRS: Gastrointestinal Symptom Rating Scale
HRQL: health-related quality of life
IBS: irritable bowel syndrome
IBS-QOL: IBS Quality of Life Impairment
mHealth: mobile health
PHQ-9: Patient Health Questionnaire-9
RCT: randomized controlled trial
VSI: Visceral Sensitivity Index
