# Supplementary material for: Efficacy of Zemedy, a Mobile Digital Therapeutic for the Self-management of Irritable Bowel Syndrome: Crossover Randomized Controlled Trial
Source: JMIR Mhealth Uhealth. 2021 May 20;9(5):e26152. doi: 10.2196/26152 (PMC8176342; doi:10.2196/26152)
Supplement: Multimedia Appendix 1 [file mhealth_v9i5e26152_app1.docx]

**Consent Form**

**Acceptability and Efficacy of Zemedy App for IBS**

Principal Investigator:

Melissa Hunt, Ph.D.

425 S. University Ave.

Philadelphia, PA 19104-6018

Office: 215-898-6478

24 Hour Emergency: 610-529-8055

e-mail: mhunt@psych.upenn.edu

**Invitation**: You are being invited to participate in a research study of a self-help treatment (the Zemedy phone app) for irritable bowel syndrome (IBS) because you have been diagnosed with IBS by a medical professional, and don’t have another serious GI disorder (like Crohn’s disease or ulcerative colitis). You must be at least 18 years of age or older to participate, and you must own a smart phone and be willing to download the Zemedy App at no cost.

**Purpose**: The purpose of this research is to determine whether a self-help app, based on a number of empirically supported treatments for IBS including cognitive-behavioral therapy, gut directed hypnotherapy, exercise, relaxation, stress management and dietary management can be an effective treatment for IBS.

**Procedures**: First, we will ask you to complete a number of baseline demographic, mood and symptom questionnaires so that we can get a good sense of how severe your IBS is, how much it is impacting your life, and how it affects your mood. At any time, you may skip any questions that make you uncomfortable. Once the investigators receive this data, they will contact you directly via e-mail within 24 business hours, informing you whether or not you are eligible to be in the study. Everyone who meets the study criteria will be assigned to one of two groups at random (like flipping a coin) - the immediate treatment group or the wait-list group. The e-mail will tell you which group you have been assigned to.

If you are assigned to the **wait-list**, we will ask you to continue with whatever you’ve been doing to manage your IBS without changing anything dramatically (like starting a new medication or going on a restrictive diet). After 8 weeks, we will ask you to complete the symptom measures again. When we get those measures, you will be offered the chance to get the app and start working through it. Once you get the app, the study will proceed as outlined in the next paragraph on immediate treatment.

If you are in the **immediate treatment** group, your first email from the investigators will contain instructions for downloading the app to your phone. During the treatment phase, you will be asked to complete a number of tasks, including:

1. Reading educational materials about IBS.

2. Tracking your IBS symptoms.

3. Practicing relaxation exercises.

4. Keeping records of your thoughts and feelings in situations in which you experienced IBS symptoms.

5. Learning new ways of thinking about social and work situations, especially about how they are impacted by your IBS symptoms.

6. Practicing new skills (e.g. relaxation, new ways of thinking) in situations you might usually prefer to avoid.

7. Learning about diet and exercise management strategies.

8. Using imagery to think about IBS symptoms differently

Your IBS symptom severity will be measured again after you have spent 8 weeks using the app. We will also ask for your feedback on the app – how user friendly it felt, how much you liked it, and so on, including any suggestions you have to make it better. Three months later, you will be contacted one last time via e-mail and will be asked to complete the symptom severity measures one last time.

**Risks**:

There are some risks to taking part in this study. You may find yourself more aware of your physical sensations and IBS symptoms for a period of time after using the app. You will also be asked to think in new ways about problems and situations that might come up in your life, including your IBS itself. Sometimes it can be uncomfortable or even scary to think about things differently, or to practice new skills. One of the questionnaires in the study will ask about symptoms of depression. If you indicate that you are severely depressed or actively thinking about suicide, the study PI (who is a licensed clinical psychologist) will reach out to you via email to check on your safety and offer you referrals to local mental health services if desired.

**Benefits**:

There is no promise of direct benefit to you for participating in this study. It is possible that this self-help intervention will help reduce your Irritable Bowel Syndrome symptoms and improve your quality of life.

**Compensation**:

If you are in the immediate treatment group, you will be compensated $20 for completing the post-treatment follow-up questionnaires in the week or so after you complete the program. You will be compensated a further $20 for completing the follow-up questionnaires 3 months after completing the program. Thus, your total compensation could be as much as $40.

If you are in the waitlist control group, you will be compensated $20 for completing the follow-up questionnaires 8 weeks after the initial questionnaires. You will be compensated a further $20 for completing another set of follow-up questionnaires in the week or so after you complete the program. Finally, you will be compensated a further $20 for completing the follow-up questionnaires 3 months after completing the program. Thus, your total compensation could be as much as $60. People who are randomly assigned to the waitlist control group can be compensated a bit more money to help make up for the fact that they had to wait for two months to access the app, and have to fill out the questionnaires one extra time.

All compensation will be in the form of Amazon.com gift vouchers that can be spent on anything you like within the Amazon system.

**Conflict of Interest:**

The originators of the Zemedy App could make money from sales of the app in the future. However, the Principal Investigator, Dr. Hunt, has no financial interest in this product, and does not stand to profit from it.

**Confidentiality**:

Use of the Zemedy application is covered by the Bold Health Privacy Policy and Terms of Use. All information collected in this study (e.g. questionnaires about your symptoms) will be kept strictly confidential, except as may be required by law.

Your information will be de-identified. De-identified means that all identifiers (like name and email address) have been removed. The information could be stored and shared for future research in this de-identified fashion. It would not be possible for future researchers to identify you as we would not share any identifiable information about you with future researchers. This can be done without again seeking your consent in the future, as permitted by law.

Your participation in this study is voluntary, and you may withdraw at any time, or skip questions you do not wish to answer. The alternative to participating in this research study is not to participate. If you wish further information regarding your rights as a research subject, you may contact the Director of Regulatory Affairs at the University of Pennsylvania by telephoning 215-898-2614. Upon clicking the “I consent” button below, you acknowledge that you have read and understood this consent form and that you agree to participate in this research study.

**By clicking on the “I consent” button below you are agreeing to take part in this research study. If you have any questions or there is something you do not understand, feel free to contact us by phone or e-mail as listed above.**
