# Supplementary material for: Efficacy of Zemedy, a Mobile Digital Therapeutic for the Self-management of Irritable Bowel Syndrome: Crossover Randomized Controlled Trial
Source: JMIR Mhealth Uhealth. 2021 May 20;9(5):e26152. doi: 10.2196/26152 (PMC8176342; doi:10.2196/26152)
Supplement: Multimedia Appendix 2 [file mhealth_v9i5e26152_app2.pdf]

CONSORT-EHEALTH (V 1.6.1) - Submission/Publication Form

The CONSORT-EHEALTH checklist is intended for authors of randomized trials evaluating web-based and Internet-based applications/interventions, including mobile interventions, electronic games (incl multiplayer games), social media, certain telehealth applications, and other interactive and/or networked electronic applications. Some of the items (e.g. all subitems under item 5 - description of the intervention) may also be applicable for other study designs.

The goal of the CONSORT EHEALTH checklist and guideline is to be
a) a guide for reporting for authors of RCTs,
b) to form a basis for appraisal of an ehealth trial (in terms of validity)

CONSORT-EHEALTH items/subitems are MANDATORY reporting items for studies published in the Journal of Medical Internet Research and other journals / scientific societies endorsing the checklist.

Items numbered 1., 2., 3., 4a., 4b etc are original CONSORT or CONSORT-NPT (non-pharmacologic treatment) items.
Items with Roman numerals (i., ii, iii, iv etc.) are CONSORT-EHEALTH extensions/clarifications.

As the CONSORT-EHEALTH checklist is still considered in a formative stage, we would ask that you also RATE ON A SCALE OF 1-5 how important/useful you feel each item is FOR THE PURPOSE OF THE CHECKLIST and reporting guideline (optional).

Mandatory reporting items are marked with a red *.
In the textboxes, either copy & paste the relevant sections from your manuscript into this form - please include any quotes from your manuscript in QUOTATION MARKS,
or answer directly by providing additional information not in the manuscript, or elaborating on why the item was not relevant for this study.

YOUR ANSWERS WILL BE PUBLISHED AS A SUPPLEMENTARY FILE TO YOUR PUBLICATION IN JMIR AND ARE CONSIDERED PART OF YOUR PUBLICATION (IF ACCEPTED).
Please fill in these questions diligently. Information will not be copyedited, so please use proper spelling and grammar, use correct capitalization, and avoid abbreviations.

DO NOT FORGET TO SAVE AS PDF _AND_ CLICK THE SUBMIT BUTTON SO YOUR ANSWERS ARE IN OUR DATABASE !!!

Citation Suggestion (if you append the pdf as Appendix we suggest to cite this paper in the caption):
Eysenbach G, CONSORT-EHEALTH Group
CONSORT-EHEALTH: Improving and Standardizing Evaluation Reports of Web-based and Mobile Health Interventions
J Med Internet Res 2011;13(4):e126
URL: http://www.jmir.org/2011/4/e126/

doi: 10.2196/jmir.1923
PMID: 22209829

* Required

Your name *

First Last

Melissa Hunt

Primary Affiliation (short), City, Country *

University of Toronto, Toronto, Canada

University of Pennsylvania, Philadelphia, Penns

Your e-mail address *

abc@gmail.com

mhunt@psych.upenn.edu

Title of your manuscript *

Provide the (draft) title of your manuscript.

The Efficacy of Zemedy, a Mobile Digital Therapeutic for the Self-Management of Irritable Bowel Syndrome: a Cross-Over, Randomized Controlled Trial

Name of your App/Software/Intervention *

If there is a short and a long/alternate name, write the short name first and add the long name in brackets.

Zemedy

Evaluated Version (if any)

e.g. "V1", "Release 2017-03-01", "Version 2.0.27913"

Zemedy 1.0

Language(s) *

What language is the intervention/app in? If multiple languages are available, separate by comma (e.g. "English, French")

English

URL of your Intervention Website or App

e.g. a direct link to the mobile app on app in appstore (itunes, Google Play), or URL of the website. If the intervention is a DVD or hardware, you can also link to an Amazon page.

https://www.zemedy.com/

URL of an image/screenshot (optional)

Your answer

Accessibility *

Can an enduser access the intervention presently?

- ○ access is free and open
- ○ access only for special usergroups, not open
- ○ access is open to everyone, but requires payment/subscription/in-app purchases
- ○ app/intervention no longer accessible
- ◉ Other: The app has been updated and only Zemedy 2.0 is now available: acc

Primary Medical Indication/Disease/Condition *

e.g. "Stress", "Diabetes", or define the target group in brackets after the condition, e.g. "Autism (Parents of children with)", "Alzheimers (Informal Caregivers of)"

Irritable bowel syndrome (IBS)

Primary Outcomes measured in trial *

comma-separated list of primary outcomes reported in the trial

IBS quality of life (IBS–QoL), Gastrointestinal S

Secondary/other outcomes

Are there any other outcomes the intervention is expected to affect?

Rome IV Questionnaire, Fear of Food Questionnaire (FFQ), Visceral sensitivity index (VSI), Gastrointestinal Cognitions Questionnaire (GI-Cog), Depression Anxiety Stress Scale (DASS), Patient Health Questionnaire - 9 (PHQ-9)

Recommended "Dose" *

What do the instructions for users say on how often the app should be used?

- ○ Approximately Daily
- ◉ Approximately Weekly
- ○ Approximately Monthly
- ○ Approximately Yearly
- ○ "as needed"
- ○ Other:

Approx. Percentage of Users (starters) still using the app as recommended after 3 months *

- (selected) unknown / not evaluated
- 0-10%
- 11-20%
- 21-30%
- 31-40%
- 41-50%
- 51-60%
- 61-70%
- 71%-80%
- 81-90%
- 91-100%
- Other:

Overall, was the app/intervention effective? *

- (selected) yes: all primary outcomes were significantly better in intervention group vs control
- partly: SOME primary outcomes were significantly better in intervention group vs control
- no statistically significant difference between control and intervention
- potentially harmful: control was significantly better than intervention in one or more outcomes
- inconclusive: more research is needed
- Other:

Article Preparation Status/Stage *

At which stage in your article preparation are you currently (at the time you fill in this form)

- ○ not submitted yet - in early draft status
- ◉ not submitted yet - in late draft status, just before submission
- ○ submitted to a journal but not reviewed yet
- ○ submitted to a journal and after receiving initial reviewer comments
- ○ submitted to a journal and accepted, but not published yet
- ○ published
- ○ Other:

Journal *

If you already know where you will submit this paper (or if it is already submitted), please provide the journal name (if it is not JMIR, provide the journal name under "other")

- ○ not submitted yet / unclear where I will submit this
- ◉ Journal of Medical Internet Research (JMIR)
- ○ JMIR mHealth and UHealth
- ○ JMIR Serious Games
- ○ JMIR Mental Health
- ○ JMIR Public Health
- ○ JMIR Formative Research
- ○ Other JMIR sister journal
- ○ Other:

Is this a full powered effectiveness trial or a pilot/feasibility trial? *

- ◯ Pilot/feasibility
- ◉ Fully powered

Manuscript tracking number *

If this is a JMIR submission, please provide the manuscript tracking number under "other" (The ms tracking number can be found in the submission acknowledgement email, or when you login as author in JMIR. If the paper is already published in JMIR, then the ms tracking number is the four-digit number at the end of the DOI, to be found at the bottom of each published article in JMIR)

- ◉ no ms number (yet) / not (yet) submitted to / published in JMIR
- ◯ Other:

TITLE AND ABSTRACT

1a) TITLE: Identification as a randomized trial in the title

1a) Does your paper address CONSORT item 1a? *

I.e does the title contain the phrase "Randomized Controlled Trial"? (if not, explain the reason under "other")

- ◉ yes
- ◯ Other:

1a-i) Identify the mode of delivery in the title

Identify the mode of delivery. Preferably use “web-based” and/or “mobile” and/or “electronic game” in the title. Avoid ambiguous terms like “online”, “virtual”, “interactive”. Use “Internet-based” only if Intervention includes non-web-based Internet components (e.g. email), use “computer-based” or “electronic” only if offline products are used. Use “virtual” only in the context of “virtual reality” (3-D worlds). Use “online” only in the context of “online support groups”. Complement or substitute product names with broader terms for the class of products (such as “mobile” or “smart phone” instead of “iphone”), especially if the application runs on different platforms.

	1	2	3	4	5	
subitem not at all important	○	○	○	○	○	essential

Does your paper address subitem 1a-i? *

Copy and paste relevant sections from manuscript title (include quotes in quotation marks "like this" to indicate direct quotes from your manuscript), or elaborate on this item by providing additional information not in the ms, or briefly explain why the item is not applicable/relevant for your study

Yes, "Mobile"

1a-ii) Non-web-based components or important co-interventions in title

Mention non-web-based components or important co-interventions in title, if any (e.g., “with telephone support”).

	1	2	3	4	5	
subitem not at all important	○	○	○	○	○	essential

Does your paper address subitem 1a-ii?

Copy and paste relevant sections from manuscript title (include quotes in quotation marks "like this" to indicate direct quotes from your manuscript), or elaborate on this item by providing additional information not in the ms, or briefly explain why the item is not applicable/relevant for your study

No non-web-based components or important co-interventions in the study

1a-iii) Primary condition or target group in the title

Mention primary condition or target group in the title, if any (e.g., “for children with Type I Diabetes”)
Example: A Web-based and Mobile Intervention with Telephone Support for Children with Type I Diabetes: Randomized Controlled Trial

	1	2	3	4	5	
subitem not at all important	○	○	○	○	○	essential

Does your paper address subitem 1a-iii? *

Copy and paste relevant sections from manuscript title (include quotes in quotation marks "like this" to indicate direct quotes from your manuscript), or elaborate on this item by providing additional information not in the ms, or briefly explain why the item is not applicable/relevant for your study

Yes, "...for the Self-Management of Irritable Bowel Syndrome"

1b) ABSTRACT: Structured summary of trial design, methods, results, and conclusions

NPT extension: Description of experimental treatment, comparator, care providers, centers, and blinding status.

1b-i) Key features/functionalities/components of the intervention and comparator in the METHODS section of the ABSTRACT

Mention key features/functionalities/components of the intervention and comparator in the abstract. If possible, also mention theories and principles used for designing the site. Keep in mind the needs of systematic reviewers and indexers by including important synonyms. (Note: Only report in the abstract what the main paper is reporting. If this information is missing from the main body of text, consider adding it)

	1	2	3	4	5	
subitem not at all important	○	○	○	○	○	essential

Does your paper address subitem 1b-i? *

Copy and paste relevant sections from the manuscript abstract (include quotes in quotation marks "like this" to indicate direct quotes from your manuscript), or elaborate on this item by providing additional information not in the ms, or briefly explain why the item is not applicable/relevant for your study

Yes, "The Zemedy app consists of 8 modules focusing on psychoeducation, relaxation training, exercise, the cognitive model of stress management, applying CBT to IBS symptoms, reducing avoidance through exposure therapy and behavioral experiments, and information about diet. Users interact with a chatbot that presents the information and encourages specific plans, homework and exercises."

1b-ii) Level of human involvement in the METHODS section of the ABSTRACT

Clarify the level of human involvement in the abstract, e.g., use phrases like "fully automated" vs. "therapist/nurse/care provider/physician-assisted" (mention number and expertise of providers involved, if any). (Note: Only report in the abstract what the main paper is reporting. If this information is missing from the main body of text, consider adding it)

	1	2	3	4	5	
subitem not at all important	○	○	○	○	○	essential

Does your paper address subitem 1b-ii?

Copy and paste relevant sections from the manuscript abstract (include quotes in quotation marks "like this" to indicate direct quotes from your manuscript), or elaborate on this item by providing additional information not in the ms, or briefly explain why the item is not applicable/relevant for your study

Yes, "The treatment was fully automated, with no therapist involvement or communication."

1b-iii) Open vs. closed, web-based (self-assessment) vs. face-to-face assessments in the METHODS section of the ABSTRACT

Mention how participants were recruited (online vs. offline), e.g., from an open access website or from a clinic or a closed online user group (closed usergroup trial), and clarify if this was a purely web-based trial, or there were face-to-face components (as part of the intervention or for assessment). Clearly say if outcomes were self-assessed through questionnaires (as common in web-based trials). Note: In traditional offline trials, an open trial (open-label trial) is a type of clinical trial in which both the researchers and participants know which treatment is being administered. To avoid confusion, use “blinded” or “unblinded” to indicated the level of blinding instead of “open”, as “open” in web-based trials usually refers to “open access” (i.e. participants can self-enrol). (Note: Only report in the abstract what the main paper is reporting. If this information is missing from the main body of text, consider adding it)

	1	2	3	4	5	
subitem not at all important	○	○	○	○	○	essential

Does your paper address subitem 1b-iii?

Copy and paste relevant sections from the manuscript abstract (include quotes in quotation marks "like this" to indicate direct quotes from your manuscript), or elaborate on this item by providing additional information not in the ms, or briefly explain why the item is not applicable/relevant for your study

Yes, "Participants were recruited online."

1b-iv) RESULTS section in abstract must contain use data

Report number of participants enrolled/assessed in each group, the use/uptake of the intervention (e.g., attrition/adherence metrics, use over time, number of logins etc.), in addition to primary/secondary outcomes. (Note: Only report in the abstract what the main paper is reporting. If this information is missing from the main body of text, consider adding it)

	1	2	3	4	5	
subitem not at all important	○	○	○	○	○	essential

Does your paper address subitem 1b-iv?

Copy and paste relevant sections from the manuscript abstract (include quotes in quotation marks "like this" to indicate direct quotes from your manuscript), or elaborate on this item by providing additional information not in the ms, or briefly explain why the item is not applicable/relevant for your study

Yes, "Scores on the GSRS, IBS-QoL, GI-COG, and VSI all improved significantly more in the treatment group [$F(1,79) = 20.49$, $P < .001$, Cohen's $d = 1.01$; $F(1,79) = 20.12$, $P < .001$, $d = 1.25$; $F(1,79) = 34.71$, $P < .001$, $d = 1.47$ and $F(1,79) = 18.7$, $P < .001$, $d = 1.07$]. Fear of food also decreased for the treatment group relative to the control group [$F(1,79) = 12.13$, $P = .001$, $d = .62$]. Depression improved significantly as measured by both the PHQ9 [$F(1,79) = 10.5$, $P = .002$, $d = 1.07$] and the DASS Depression Subscale [$F(1,79) = 6.03$, $P = .016$, $d = .83$], as did the stress subscale of the DASS [$F(1,79) = 4.47$, $P = .04$, $d = .65$] in the completer analysis but not the intent-to-treat analysis."

1b-v) CONCLUSIONS/DISCUSSION in abstract for negative trials

Conclusions/Discussions in abstract for negative trials: Discuss the primary outcome - if the trial is negative (primary outcome not changed), and the intervention was not used, discuss whether negative results are attributable to lack of uptake and discuss reasons. (Note: Only report in the abstract what the main paper is reporting. If this information is missing from the main body of text, consider adding it)

	1	2	3	4	5	
subitem not at all important	○	○	○	○	○	essential

Does your paper address subitem 1b-v?

Copy and paste relevant sections from the manuscript abstract (include quotes in quotation marks "like this" to indicate direct quotes from your manuscript), or elaborate on this item by providing additional information not in the ms, or briefly explain why the item is not applicable/relevant for your study

No, the outcomes of the trial were not negative in our study. We reported improvement on the primary outcome measures.

INTRODUCTION

2a) In INTRODUCTION: Scientific background and explanation of rationale

2a-i) Problem and the type of system/solution

Describe the problem and the type of system/solution that is object of the study: intended as stand-alone intervention vs. incorporated in broader health care program? Intended for a particular patient population? Goals of the intervention, e.g., being more cost-effective to other interventions, replace or complement other solutions? (Note: Details about the intervention are provided in “Methods” under 5)

	1	2	3	4	5	
subitem not at all important	○	○	○	○	○	essential

Does your paper address subitem 2a-i? *

Copy and paste relevant sections from the manuscript (include quotes in quotation marks "like this" to indicate direct quotes from your manuscript), or elaborate on this item by providing additional information not in the ms, or briefly explain why the item is not applicable/relevant for your study

Yes, "Many studies have demonstrated that IBS has high rates of psychiatric comorbidity (up to 90% in treatment seeking patients) [1,2], and causes social and occupational impairment [3]. Beyond the core symptoms of abdominal pain and altered bowel habits, individuals with IBS suffer from a host of related difficulties that substantially impair health-related quality of life and functioning." "While CBT is a promising treatment, access to IBS-specific CBT remains low for patients. There is a lack of clinicians competent in delivering GI-specific CBT [3]. Additionally, the cost of treatment looms high; individuals often lack insurance coverage for psychotherapy and must pay out of pocket, which can be burdensome given the hundreds of dollars their IBS likely already costs them [18]. It is therefore necessary to develop a cheaper, more easily accessible alternative mode of treatment."

2a-ii) Scientific background, rationale: What is known about the (type of) system

Scientific background, rationale: What is known about the (type of) system that is the object of the study (be sure to discuss the use of similar systems for other conditions/diagnoses, if appropiate), motivation for the study, i.e. what are the reasons for and what is the context for this specific study, from which stakeholder viewpoint is the study performed, potential impact of findings [2]. Briefly justify the choice of the comparator.

	1	2	3	4	5	
subitem not at all important	○	○	○	○	○	essential

Does your paper address subitem 2a-ii? *

Copy and paste relevant sections from the manuscript (include quotes in quotation marks "like this" to indicate direct quotes from your manuscript), or elaborate on this item by providing additional information not in the ms, or briefly explain why the item is not applicable/relevant for your study

Yes, "Over the past two decades, cognitive behavioral therapy (CBT) has repeatedly proven to be an efficacious treatment for individuals suffering from IBS [10, 11]. Specifically, there is empirical support that CBT reduces GI symptom severity and impairment to quality of life [12, 13]." "Many groups have tested variants of CBT for IBS with limited or distant therapist involvement (e.g., via email) [19, 15] and typically obtain robust effect sizes. Everitt et. al. found that web-based and telephone-based CBT improved IBS more than treatment as usual [20]. Several treatment manuals and self-help books are available that detail the CBT treatment approach, and one [21] was found to be efficacious as a stand alone treatment in a randomized controlled trial [22]." "CBT is among the forms of treatment increasingly being delivered via apps. In their review of eight CBT apps, Rathbone, Clarry and Prescott found that CBT self-help apps can be efficacious, most notably in alleviating depressive symptoms [24]."

2b) In INTRODUCTION: Specific objectives or hypotheses

Does your paper address CONSORT subitem 2b? *

Copy and paste relevant sections from the manuscript (include quotes in quotation marks "like this" to indicate direct quotes from your manuscript), or elaborate on this item by providing additional information not in the ms, or briefly explain why the item is not applicable/relevant for your study

Yes, "The purpose of this study was to test the acceptability and efficacy of a novel digital app (Zemedy) that applies CBT to IBS."

METHODS

3a) Description of trial design (such as parallel, factorial) including allocation ratio

Does your paper address CONSORT subitem 3a? *

Copy and paste relevant sections from the manuscript (include quotes in quotation marks "like this" to indicate direct quotes from your manuscript), or elaborate on this item by providing additional information not in the ms, or briefly explain why the item is not applicable/relevant for your study

Yes, "This was a randomized, wait-list control, cross-over trial with assessments conducted at baseline, post-intervention (8 weeks), post cross over to intervention for the wait-list control group, and at follow-up (3 months). After completing the consent and all the baseline measures, participants were randomly allocated by a research coordinator to either immediate treatment or waitlist control using the coin toss function of random.org.", and "Participants who met the inclusion and exclusion criteria were allocated to condition using the coin toss feature of random.org. A total of 62 participants were assigned to the immediate treatment condition and 59 were assigned to the waitlist control."

3b) Important changes to methods after trial commencement (such as eligibility criteria), with reasons

Does your paper address CONSORT subitem 3b? *

Copy and paste relevant sections from the manuscript (include quotes in quotation marks "like this" to indicate direct quotes from your manuscript), or elaborate on this item by providing additional information not in the ms, or briefly explain why the item is not applicable/relevant for your study

No, there were no important changes to methods after trial commencement

3b-i) Bug fixes, Downtimes, Content Changes

Bug fixes, Downtimes, Content Changes: ehealth systems are often dynamic systems. A description of changes to methods therefore also includes important changes made on the intervention or comparator during the trial (e.g., major bug fixes or changes in the functionality or content) (5-iii) and other “unexpected events” that may have influenced study design such as staff changes, system failures/downtimes, etc. [2].

	1	2	3	4	5	
subitem not at all important	○	○	○	○	○	essential

Does your paper address subitem 3b-i?

Copy and paste relevant sections from the manuscript (include quotes in quotation marks "like this" to indicate direct quotes from your manuscript), or elaborate on this item by providing additional information not in the ms, or briefly explain why the item is not applicable/relevant for your study

No “unexpected events” influenced study design

4a) Eligibility criteria for participants

Does your paper address CONSORT subitem 4a? *

Copy and paste relevant sections from the manuscript (include quotes in quotation marks "like this" to indicate direct quotes from your manuscript), or elaborate on this item by providing additional information not in the ms, or briefly explain why the item is not applicable/relevant for your study

Yes, "Inclusion criteria consisted of being 18 years of age or older, and reporting having been previously diagnosed by a physician with IBS. Owning a smartphone and computer/internet literacy were de facto eligibility criteria.

Exclusion criteria consisted of having another comorbid GI disorder, such as celiac disease or an inflammatory bowel disease. Exclusion criteria also included severe depression and/or suicidal ideation - defined as a score of 20 or above on the Patient Health Questionnaire - 9 (PHQ-9) and/or positive endorsement at the level of 2 or 3 on the suicide question (item 9) of the Beck Depression Inventory. Twenty-five individuals met this criterion. They were excluded from the trial, but were given immediate access to the app."

4a-i) Computer / Internet literacy

Computer / Internet literacy is often an implicit “de facto” eligibility criterion - this should be explicitly clarified.

	1	2	3	4	5	
subitem not at all important	○	○	○	○	○	essential

Does your paper address subitem 4a-i?

Copy and paste relevant sections from the manuscript (include quotes in quotation marks "like this" to indicate direct quotes from your manuscript), or elaborate on this item by providing additional information not in the ms, or briefly explain why the item is not applicable/relevant for your study

Yes, "Owning a smartphone and computer/internet literacy were de facto eligibility criteria."

4a-ii) Open vs. closed, web-based vs. face-to-face assessments:

Open vs. closed, web-based vs. face-to-face assessments: Mention how participants were recruited (online vs. offline), e.g., from an open access website or from a clinic, and clarify if this was a purely web-based trial, or there were face-to-face components (as part of the intervention or for assessment), i.e., to what degree got the study team to know the participant. In online-only trials, clarify if participants were quasi-anonymous and whether having multiple identities was possible or whether technical or logistical measures (e.g., cookies, email confirmation, phone calls) were used to detect/prevent these.

	1	2	3	4	5	
subitem not at all important	○	○	○	○	○	essential

Does your paper address subitem 4a-ii? *

Copy and paste relevant sections from the manuscript (include quotes in quotation marks "like this" to indicate direct quotes from your manuscript), or elaborate on this item by providing additional information not in the ms, or briefly explain why the item is not applicable/relevant for your study

Yes, "Participants were recruited for the trial through IBS specific social media sites with a combination of graphic advertisements and posts and comments on threads informing site users about the study. Most participants came to the study through Facebook (N=30), Twitter (N=32), and the IBS subReddit (N=51). There were no face-to-face components to the trial in either recruitment, assessment or intervention."

4a-iii) Information giving during recruitment

Information given during recruitment. Specify how participants were briefed for recruitment and in the informed consent procedures (e.g., publish the informed consent documentation as appendix, see also item X26), as this information may have an effect on user self-selection, user expectation and may also bias results.

	1	2	3	4	5	
subitem not at all important	O	O	O	O	O	essential

Does your paper address subitem 4a-iii?

Copy and paste relevant sections from the manuscript (include quotes in quotation marks "like this" to indicate direct quotes from your manuscript), or elaborate on this item by providing additional information not in the ms, or briefly explain why the item is not applicable/relevant for your study

Yes, "Posts and advertisements included a link to a secure University of Pennsylvania Qualtrics study page. On following the link, potential participants would first see the detailed explanation of the research (Consent Form - See Appendix A) and would consent to completing the baseline questionnaires. Questionnaires were completed via Qualtrics and could be downloaded securely by the research team. Participants were identified by email during data collection. All data was stored de-identified. All recruitment and follow-up occurred between 10/01/2019 and 11/01/2020. The trial ended upon successful completion."

4b) Settings and locations where the data were collected

Does your paper address CONSORT subitem 4b? *

Copy and paste relevant sections from the manuscript (include quotes in quotation marks "like this" to indicate direct quotes from your manuscript), or elaborate on this item by providing additional information not in the ms, or briefly explain why the item is not applicable/relevant for your study

Yes, "Questionnaires were completed via Qualtrics and could be downloaded securely by the research team. Participants were identified by email during data collection. All data was stored de-identified."

4b-i) Report if outcomes were (self-)assessed through online questionnaires

Clearly report if outcomes were (self-)assessed through online questionnaires (as common in web-based trials) or otherwise.

	1	2	3	4	5	
subitem not at all important	○	○	○	○	○	essential

Does your paper address subitem 4b-i? *

Copy and paste relevant sections from the manuscript (include quotes in quotation marks "like this" to indicate direct quotes from your manuscript), or elaborate on this item by providing additional information not in the ms, or briefly explain why the item is not applicable/relevant for your study

Yes, "Moreover, all outcome data was self-report."

4b-ii) Report how institutional affiliations are displayed

Report how institutional affiliations are displayed to potential participants [on ehealth media], as affiliations with prestigious hospitals or universities may affect volunteer rates, use, and reactions with regards to an intervention.(Not a required item – describe only if this may bias results)

	1	2	3	4	5	
subitem not at all important	○	○	○	○	○	essential

Does your paper address subitem 4b-ii?

Copy and paste relevant sections from the manuscript (include quotes in quotation marks "like this" to indicate direct quotes from your manuscript), or elaborate on this item by providing additional information not in the ms, or briefly explain why the item is not applicable/relevant for your study

Yes, "Posts and advertisements included a link to a secure University of Pennsylvania Qualtrics study page."

5) The interventions for each group with sufficient details to allow replication, including how and when they were actually administered

5-i) Mention names, credential, affiliations of the developers, sponsors, and owners

Mention names, credential, affiliations of the developers, sponsors, and owners [6] (if authors/evaluators are owners or developer of the software, this needs to be declared in a “Conflict of interest” section or mentioned elsewhere in the manuscript).

	1	2	3	4	5	
subitem not at all important	○	○	○	○	○	essential

Does your paper address subitem 5-i?

Copy and paste relevant sections from the manuscript (include quotes in quotation marks "like this" to indicate direct quotes from your manuscript), or elaborate on this item by providing additional information not in the ms, or briefly explain why the item is not applicable/relevant for your study

Yes, "Zemedy 1.0 is a mobile phone application designed by Bold Health, a UK-based digital health company, in collaboration with Melissa Hunt based on her empirically supported self-help book."

5-ii) Describe the history/development process

Describe the history/development process of the application and previous formative evaluations (e.g., focus groups, usability testing), as these will have an impact on adoption/use rates and help with interpreting results.

	1	2	3	4	5	
subitem not at all important	○	○	○	○	○	essential

Does your paper address subitem 5-ii?

Copy and paste relevant sections from the manuscript (include quotes in quotation marks "like this" to indicate direct quotes from your manuscript), or elaborate on this item by providing additional information not in the ms, or briefly explain why the item is not applicable/relevant for your study

Yes, "Zemedy 1.0 is a mobile phone application designed by Bold Health, a UK-based digital health company, in collaboration with Melissa Hunt based on her empirically supported self-help book [21]." The reference (Hunt, M. G. (2016). Reclaim Your Life from IBS: A Scientifically Proven Plan for Relief without Restrictive Diets. Sterling, NY, NY. ISBN-10: 145491887X) is the book on which the CBT protocol is based, whereas the Bold Health team was behind the conversion of the CBT protocol into the digital format, coordinating the team of programmers and designers who created the app itself.

5-iii) Revisions and updating

Revisions and updating. Clearly mention the date and/or version number of the application/intervention (and comparator, if applicable) evaluated, or describe whether the intervention underwent major changes during the evaluation process, or whether the development and/or content was “frozen” during the trial. Describe dynamic components such as news feeds or changing content which may have an impact on the replicability of the intervention (for unexpected events see item 3b).

	1	2	3	4	5	
subitem not at all important	○	○	○	○	○	essential

Does your paper address subitem 5-iii?

Copy and paste relevant sections from the manuscript (include quotes in quotation marks "like this" to indicate direct quotes from your manuscript), or elaborate on this item by providing additional information not in the ms, or briefly explain why the item is not applicable/relevant for your study

Yes, "Zemedy 1.0 is a mobile phone application designed by Bold Health, a UK-based digital health company, in collaboration with Melissa Hunt based on her empirically supported self-help book." No revisions and updating occurred during the evaluation process, nor was content "frozen" during the trial, nor was there a comparator in the study. Additionally, no dynamic components were featured in the app in Version 1.0.

5-iv) Quality assurance methods

Provide information on quality assurance methods to ensure accuracy and quality of information provided [1], if applicable.

	1	2	3	4	5	
subitem not at all important	○	○	○	○	○	essential

Does your paper address subitem 5-iv?

Copy and paste relevant sections from the manuscript (include quotes in quotation marks "like this" to indicate direct quotes from your manuscript), or elaborate on this item by providing additional information not in the ms, or briefly explain why the item is not applicable/relevant for your study

Your answer

5-v) Ensure replicability by publishing the source code, and/or providing screenshots/screen-capture video, and/or providing flowcharts of the algorithms used

Ensure replicability by publishing the source code, and/or providing screenshots/screen-capture video, and/or providing flowcharts of the algorithms used. Replicability (i.e., other researchers should in principle be able to replicate the study) is a hallmark of scientific reporting.

	1	2	3	4	5	
subitem not at all important	○	○	○	○	○	essential

Does your paper address subitem 5-v?

Copy and paste relevant sections from the manuscript (include quotes in quotation marks "like this" to indicate direct quotes from your manuscript), or elaborate on this item by providing additional information not in the ms, or briefly explain why the item is not applicable/relevant for your study

Yes, "See (Figure 1) for screenshots of the Zemedy app."

5-vi) Digital preservation

Digital preservation: Provide the URL of the application, but as the intervention is likely to change or disappear over the course of the years; also make sure the intervention is archived (Internet Archive, webcitation.org, and/or publishing the source code or screenshots/videos alongside the article). As pages behind login screens cannot be archived, consider creating demo pages which are accessible without login.

	1	2	3	4	5	
subitem not at all important	○	○	○	○	○	essential

Does your paper address subitem 5-vi?

Copy and paste relevant sections from the manuscript (include quotes in quotation marks "like this" to indicate direct quotes from your manuscript), or elaborate on this item by providing additional information not in the ms, or briefly explain why the item is not applicable/relevant for your study

Your answer

5-vii) Access

Access: Describe how participants accessed the application, in what setting/context, if they had to pay (or were paid) or not, whether they had to be a member of specific group. If known, describe how participants obtained "access to the platform and Internet" [1]. To ensure access for editors/reviewers/readers, consider to provide a "backdoor" login account or demo mode for reviewers/readers to explore the application (also important for archiving purposes, see vi).

	1	2	3	4	5	
subitem not at all important	○	○	○	○	○	essential

Does your paper address subitem 5-vii? *

Copy and paste relevant sections from the manuscript (include quotes in quotation marks "like this" to indicate direct quotes from your manuscript), or elaborate on this item by providing additional information not in the ms, or briefly explain why the item is not applicable/relevant for your study

Yes, "Participants were provided with a link to download the application. They were provided the application at no cost. The entire intervention was delivered within the app with no human involvement (e.g. therapist guidance or feedback). If participants experienced technical difficulties they could reach out to tech support at Bold Health. They received a single email at 4 weeks from a research coordinator in the trial providing general encouragement to continue working through the app (if they were in the immediate treatment group) or to "hang in there" (if they were in the waitlist control group)."

5-viii) Mode of delivery, features/functionalities/components of the intervention and comparator, and the theoretical framework

Describe mode of delivery, features/functionalities/components of the intervention and comparator, and the theoretical framework [6] used to design them (instructional strategy [1], behaviour change techniques, persuasive features, etc., see e.g., [7, 8] for terminology). This includes an in-depth description of the content (including where it is coming from and who developed it) [1]," whether [and how] it is tailored to individual circumstances and allows users to track their progress and receive feedback" [6]. This also includes a description of communication delivery channels and – if computer-mediated communication is a component – whether communication was synchronous or asynchronous [6]. It also includes information on presentation strategies [1], including page design principles, average amount of text on pages, presence of hyperlinks to other resources, etc. [1].

	1	2	3	4	5	
subitem not at all important	○	○	○	○	○	essential

Does your paper address subitem 5-viii? *

Copy and paste relevant sections from the manuscript (include quotes in quotation marks "like this" to indicate direct quotes from your manuscript), or elaborate on this item by providing additional information not in the ms, or briefly explain why the item is not applicable/relevant for your study

Yes, "Zemedy 1.0 is a mobile phone application designed by Bold Health, a UK-based digital health company, in collaboration with Melissa Hunt based on her empirically supported self-help book [21]. The app treats irritable bowel syndrome through cognitive behavioral therapy specifically developed for IBS. Users of either iOS or Android smartphones are guided through the app by a chatbot with whom they "text." The app consists of ten modules. The first two modules are devoted to psychoeducation about the etiology of IBS and CBT's effectiveness in treating it. The remaining eight modules teach users about various CBT strategies to mitigate the impact of IBS on daily life, including relaxation training, exercise, cognitive-restructuring and de-catastrophizing, exposure exercises to reduce avoidance, and behavioral experiments. It also encourages a healthful (but not highly restrictive) diet. See (Figure 1) for screenshots of the Zemedy app. Users are prompted to apply these strategies to their daily lives. Similar to the chronic pain treatment developed by Hauser-Ulrich, et al. (2020), it is designed to be completed in 8 weeks [29]. Participants were encouraged to read through the first 5 modules (education, relaxation training and exercise) in the first week, and to practice relaxation exercises daily. The remaining modules were designed to be worked through approximately one per week, with practice and homework exercises done daily to learn and apply the skills.

The app also includes a "flare module," which users can access at any point to address immediate GI pain and anxiety. Shah et. al. found that mind body interventions, such as relaxation training and hypnosis, have moderate effect sizes in reducing IBS symptoms [14]. The flare module contains a variety of exercises such as deep breathing, progressive muscle relaxation, relaxation imagery, and hypnotherapy scripts that help mitigate distress and discomfort in the moment."

5-ix) Describe use parameters

Describe use parameters (e.g., intended "doses" and optimal timing for use). Clarify what instructions or recommendations were given to the user, e.g., regarding timing, frequency, heaviness of use, if any, or was the intervention used ad libitum.

	1	2	3	4	5	
subitem not at all important	○	○	○	○	○	essential

Does your paper address subitem 5-ix?

Copy and paste relevant sections from the manuscript (include quotes in quotation marks "like this" to indicate direct quotes from your manuscript), or elaborate on this item by providing additional information not in the ms, or briefly explain why the item is not applicable/relevant for your study

Dosage was not captured in our study. However, "The app consists of ten modules. The first two modules are devoted to psychoeducation about the etiology of IBS and CBT's effectiveness in treating it. The remaining eight modules teach users about various CBT strategies to mitigate the impact of IBS on daily life, including relaxation training, exercise, cognitive-restructuring and de-catastrophizing, exposure exercises to reduce avoidance, and behavioral experiments. It also encourages a healthful (but not highly restrictive) diet. See (Figure 1) for screenshots of the Zemedy app. Users are prompted to apply these strategies to their daily lives. Similar to the chronic pain treatment developed by Hauser-Ulrich, et al. (2020), it is designed to be completed in 8 weeks [29]. Participants were encouraged to read through the first 5 modules (education, relaxation training and exercise) in the first week, and to practice relaxation exercises daily. The remaining modules were designed to be worked through approximately one per week, with practice and homework exercises done daily to learn and apply the skills."

5-x) Clarify the level of human involvement

Clarify the level of human involvement (care providers or health professionals, also technical assistance) in the e-intervention or as co-intervention (detail number and expertise of professionals involved, if any, as well as "type of assistance offered, the timing and frequency of the support, how it is initiated, and the medium by which the assistance is delivered". It may be necessary to distinguish between the level of human involvement required for the trial, and the level of human involvement required for a routine application outside of a RCT setting (discuss under item 21 – generalizability).

	1	2	3	4	5	
subitem not at all important	○	○	○	○	○	essential

Does your paper address subitem 5-x?

Copy and paste relevant sections from the manuscript (include quotes in quotation marks "like this" to indicate direct quotes from your manuscript), or elaborate on this item by providing additional information not in the ms, or briefly explain why the item is not applicable/relevant for your study

Yes, " The entire intervention was delivered within the app with no human involvement (e.g. therapist guidance or feedback). If participants experienced technical difficulties they could reach out to tech support at Bold Health. They received a single email at 4 weeks from a research coordinator in the trial providing general encouragement to continue working through the app (if they were in the immediate treatment group) or to “hang in there” (if they were in the waitlist control group)."

5-xi) Report any prompts/reminders used

Report any prompts/reminders used: Clarify if there were prompts (letters, emails, phone calls, SMS) to use the application, what triggered them, frequency etc. It may be necessary to distinguish between the level of prompts/reminders required for the trial, and the level of prompts/reminders for a routine application outside of a RCT setting (discuss under item 21 – generalizability).

	1	2	3	4	5	
subitem not at all important	○	○	○	○	○	essential

Does your paper address subitem 5-xi? *

Copy and paste relevant sections from the manuscript (include quotes in quotation marks "like this" to indicate direct quotes from your manuscript), or elaborate on this item by providing additional information not in the ms, or briefly explain why the item is not applicable/relevant for your study

Yes, "They [the participants] received a single email at 4 weeks from a research coordinator in the trial providing general encouragement to continue working through the app (if they were in the immediate treatment group) or to “hang in there” (if they were in the waitlist control group)."

5-xii) Describe any co-interventions (incl. training/support)

Describe any co-interventions (incl. training/support): Clearly state any interventions that are provided in addition to the targeted eHealth intervention, as ehealth intervention may not be designed as stand-alone intervention. This includes training sessions and support [1]. It may be necessary to distinguish between the level of training required for the trial, and the level of training for a routine application outside of a RCT setting (discuss under item 21 – generalizability.

	1	2	3	4	5	
subitem not at all important	○	○	○	○	○	essential

Does your paper address subitem 5-xii? *

Copy and paste relevant sections from the manuscript (include quotes in quotation marks "like this" to indicate direct quotes from your manuscript), or elaborate on this item by providing additional information not in the ms, or briefly explain why the item is not applicable/relevant for your study

No co-interventions are used alongside Zemedy.

6a) Completely defined pre-specified primary and secondary outcome measures, including how and when they were assessed

Does your paper address CONSORT subitem 6a? *

Copy and paste relevant sections from the manuscript (include quotes in quotation marks "like this" to indicate direct quotes from your manuscript), or elaborate on this item by providing additional information not in the ms, or briefly explain why the item is not applicable/relevant for your study

Yes, "The IBS–QOL is a 34 item, self-report measure specific to IBS designed to assess the impact of IBS on quality of life", "The GSRS-IBS contains 13 self-report items rated on a 6-point Likert scale ranging from 1 (no discomfort at all) to 7 (very severe discomfort) [33]. Total scores range from 13 to 91.", "We used a questionnaire to determine whether participants met current Rome IV diagnostic criteria for IBS. Our questionnaire was based on the Rome IV IBS-specific Questionnaire, which is a validated self-report scale that covers the diagnostic criteria for IBS." "The FFQ is an 18-item, self-report questionnaire that measures fear, avoidance of food, as well as life interference and loss of pleasure from eating [36]." The VSI is a unidimensional, 15-item scale that measures gastrointestinal symptom-specific anxiety [6, 37].", "The GI-Cog consists of 16 self-report items that are rated on a 5-point Likert scale, ranging from 0 (Hardly) to 4 (Very much). Individual items are summed, and total scores range from 0 to 64.", "The DASS is a 42-item, self-administered questionnaire which measures the magnitude of depression, anxiety, and stress, independently.", "The PHQ-9 is a depression scale that consists of 9 self-report items."

6a-i) Online questionnaires: describe if they were validated for online use and apply CHERRIES items to describe how the questionnaires were designed/deployed

If outcomes were obtained through online questionnaires, describe if they were validated for online use and apply CHERRIES items to describe how the questionnaires were designed/deployed [9].

	1	2	3	4	5	
subitem not at all important	○	○	○	○	○	essential

Does your paper address subitem 6a-i?

Copy and paste relevant sections from manuscript text

N/A

6a-ii) Describe whether and how “use” (including intensity of use/dosage) was defined/measured/monitored

Describe whether and how “use” (including intensity of use/dosage) was defined/measured/monitored (logins, logfile analysis, etc.). Use/adoption metrics are important process outcomes that should be reported in any ehealth trial.

	1	2	3	4	5	
subitem not at all important	O	O	O	O	O	essential

Does your paper address subitem 6a-ii?

Copy and paste relevant sections from manuscript text

Yes, "Dosage was measured according to the time of mobile app use and frequency of items completed. We calculated both factors based on mobile log data that registered the screens viewed and components used by the participants during each visit, along with the total amount of time spent on the app. Time of app use represents the overall amount of time spent on the Zemedy app. The mobile app sent usage Data to the backend system each time a participant visited the app. Data includes time and date of each session on the app."

6a-iii) Describe whether, how, and when qualitative feedback from participants was obtained

Describe whether, how, and when qualitative feedback from participants was obtained (e.g., through emails, feedback forms, interviews, focus groups).

	1	2	3	4	5	
subitem not at all important	O	O	O	O	O	essential

Does your paper address subitem 6a-iii?

Copy and paste relevant sections from manuscript text

N/A

6b) Any changes to trial outcomes after the trial commenced, with reasons

Does your paper address CONSORT subitem 6b? *

Copy and paste relevant sections from the manuscript (include quotes in quotation marks "like this" to indicate direct quotes from your manuscript), or elaborate on this item by providing additional information not in the ms, or briefly explain why the item is not applicable/relevant for your study

No changes to trial outcomes after the trial commenced. However, "Unfortunately, the onset of the COVID-19 pandemic coincided with the follow-up portion of the trial, and it is unclear the extent to which the pandemic affected both attrition and long term results." but "Three month follow-up data were collected for all subjects (both the immediate treatment group and the wait list group, who had been crossed over to treatment) between April and September of 2020. Unfortunately, this meant that all follow-up data were collected after the onset of the COVID-19 pandemic. Nevertheless, participants (all of whom had had access to the active treatment at this point) continued to show significant improvement over baseline on all outcome variables except depression."

7a) How sample size was determined

NPT: When applicable, details of whether and how the clustering by care provides or centers was addressed

7a-i) Describe whether and how expected attrition was taken into account when calculating the sample size

Describe whether and how expected attrition was taken into account when calculating the sample size.

	1	2	3	4	5	
subitem not at all important	○	○	○	○	○	essential

Does your paper address subitem 7a-i?

Copy and paste relevant sections from manuscript title (include quotes in quotation marks "like this" to indicate direct quotes from your manuscript), or elaborate on this item by providing additional information not in the ms, or briefly explain why the item is not applicable/relevant for your study

Yes, "The power analysis showed that 30 participants per randomised group, would have 85% statistical power at a two-sided significance level of 0.05 to detect an effect size of 0.76. The effect size was chosen as previous studies of internet delivered CBT for IBS had found effect sizes similar to this for health-related quality of life (HRQL) and GI symptoms outcomes [15, 22]. Assuming 50% attrition, as is common in internet-based intervention studies, we aimed to recruit 120 participants in order to have ample power to both detect main effects and to explore potential mediators and moderators. "

7b) When applicable, explanation of any interim analyses and stopping guidelines

Does your paper address CONSORT subitem 7b? *

Copy and paste relevant sections from the manuscript (include quotes in quotation marks "like this" to indicate direct quotes from your manuscript), or elaborate on this item by providing additional information not in the ms, or briefly explain why the item is not applicable/relevant for your study

N/A

8a) Method used to generate the random allocation sequence

NPT: When applicable, how care providers were allocated to each trial group

Does your paper address CONSORT subitem 8a? *

Copy and paste relevant sections from the manuscript (include quotes in quotation marks "like this" to indicate direct quotes from your manuscript), or elaborate on this item by providing additional information not in the ms, or briefly explain why the item is not applicable/relevant for your study

Yes, "After completing the consent and all the baseline measures, participants were randomly allocated by a research coordinator to either immediate treatment or waitlist control using the coin toss function of random.org. "

8b) Type of randomisation; details of any restriction (such as blocking and block size)

Does your paper address CONSORT subitem 8b? *

Copy and paste relevant sections from the manuscript (include quotes in quotation marks "like this" to indicate direct quotes from your manuscript), or elaborate on this item by providing additional information not in the ms, or briefly explain why the item is not applicable/relevant for your study

Yes, "Participants who met the inclusion and exclusion criteria were allocated to condition using the coin toss feature of random.org. A total of 62 participants were assigned to the immediate treatment condition and 59 were assigned to the waitlist control. The allocation sequence was concealed to participants until they were enrolled, had completed baseline data collection and been assigned to a group. The majority of baseline symptom severity measures were not significantly different between the immediate treatment and waitlist control groups. However, the waitlist control reported statistically significantly more depression and more impaired health related quality of life than the immediate treatment group. While the design should have yielded low risk of bias from randomization, the slight differences in symptom severity at baseline suggest some concerns about randomization, according to the Cochrane risk of bias tool."

9) Mechanism used to implement the random allocation sequence (such as sequentially numbered containers), describing any steps taken to conceal the sequence until interventions were assigned

Does your paper address CONSORT subitem 9? *

Copy and paste relevant sections from the manuscript (include quotes in quotation marks "like this" to indicate direct quotes from your manuscript), or elaborate on this item by providing additional information not in the ms, or briefly explain why the item is not applicable/relevant for your study

Yes, "Participants who met the inclusion and exclusion criteria were allocated to condition using the coin toss feature of random.org. A total of 62 participants were assigned to the immediate treatment condition and 59 were assigned to the waitlist control. The allocation sequence was concealed to participants until they were enrolled, had completed baseline data collection and been assigned to a group."

10) Who generated the random allocation sequence, who enrolled participants, and who assigned participants to interventions

Does your paper address CONSORT subitem 10? *

Copy and paste relevant sections from the manuscript (include quotes in quotation marks "like this" to indicate direct quotes from your manuscript), or elaborate on this item by providing additional information not in the ms, or briefly explain why the item is not applicable/relevant for your study

Yes, "After completing the consent and all the baseline measures, participants were randomly allocated by a research coordinator to either immediate treatment or waitlist control using the coin toss function of random.org."

11a) If done, who was blinded after assignment to interventions (for example, participants, care providers, those assessing outcomes) and how

NPT: Whether or not administering co-interventions were blinded to group assignment

11a-i) Specify who was blinded, and who wasn't

Specify who was blinded, and who wasn't. Usually, in web-based trials it is not possible to blind the participants [1, 3] (this should be clearly acknowledged), but it may be possible to blind outcome assessors, those doing data analysis or those administering co-interventions (if any).

	1	2	3	4	5	
subitem not at all important	○	○	○	○	○	essential

Does your paper address subitem 11a-i? *

Copy and paste relevant sections from the manuscript (include quotes in quotation marks "like this" to indicate direct quotes from your manuscript), or elaborate on this item by providing additional information not in the ms, or briefly explain why the item is not applicable/relevant for your study

Yes, "Because of the nature of the trial (immediate treatment versus waitlist control group), neither participants nor research coordinators were blinded to condition. However, there were no deviations from the intended intervention. Moreover, all outcome data was self-report. Thus, blinding of evaluators was neither possible nor necessary."

11a-ii) Discuss e.g., whether participants knew which intervention was the "intervention of interest" and which one was the "comparator"

Informed consent procedures (4a-ii) can create biases and certain expectations - discuss e.g., whether participants knew which intervention was the "intervention of interest" and which one was the "comparator".

	1	2	3	4	5	
subitem not at all important	○	○	○	○	○	essential

Does your paper address subitem 11a-ii?

Copy and paste relevant sections from the manuscript (include quotes in quotation marks "like this" to indicate direct quotes from your manuscript), or elaborate on this item by providing additional information not in the ms, or briefly explain why the item is not applicable/relevant for your study

Yes, "A total of 62 participants were assigned to the immediate treatment condition and 59 were assigned to the waitlist control. The allocation sequence was concealed to participants until they were enrolled, had completed baseline data collection and been assigned to a group."

11b) If relevant, description of the similarity of interventions

(this item is usually not relevant for ehealth trials as it refers to similarity of a placebo or sham intervention to a active medication/intervention)

Does your paper address CONSORT subitem 11b? *

Copy and paste relevant sections from the manuscript (include quotes in quotation marks "like this" to indicate direct quotes from your manuscript), or elaborate on this item by providing additional information not in the ms, or briefly explain why the item is not applicable/relevant for your study

N/A

12a) Statistical methods used to compare groups for primary and secondary outcomes

NPT: When applicable, details of whether and how the clustering by care providers or centers was addressed

Does your paper address CONSORT subitem 12a? *

Copy and paste relevant sections from the manuscript (include quotes in quotation marks "like this" to indicate direct quotes from your manuscript), or elaborate on this item by providing additional information not in the ms, or briefly explain why the item is not applicable/relevant for your study

Yes, "Univariate general linear models in SPSS V25 were used to examine between group effects at post treatment (8 weeks), controlling for baseline levels of the dependent variable. Paired sample t-tests were used to examine within group change over their treatment phase for each group and maintenance of gains from post treatment to 3 months follow-up. The robustness of these analyses were examined in an intent-to-treat sensitivity analysis by using multiple imputation."

12a-i) Imputation techniques to deal with attrition / missing values

Imputation techniques to deal with attrition / missing values: Not all participants will use the intervention/comparator as intended and attrition is typically high in ehealth trials. Specify how participants who did not use the application or dropped out from the trial were treated in the statistical analysis (a complete case analysis is strongly discouraged, and simple imputation techniques such as LOCF may also be problematic [4]).

	1	2	3	4	5	
subitem not at all important	○	○	○	○	○	essential

Does your paper address subitem 12a-i? *

Copy and paste relevant sections from the manuscript (include quotes in quotation marks "like this" to indicate direct quotes from your manuscript), or elaborate on this item by providing additional information not in the ms, or briefly explain why the item is not applicable/relevant for your study

Yes, "As is shown later missing data at follow-up was not entirely missing at random. Therefore, baseline outcome measures were included in the imputation model as predictors together with the follow-up set of measures with missing data and imputation using the fully conditional specification [46] conducted to create 15 imputed datasets. Regression models were then fitted as in the primary analysis, and pooled estimates of the treatment effect calculated. Three sets of imputed datasets were created, one for each follow-up data point, baseline measures included in each."

12b) Methods for additional analyses, such as subgroup analyses and adjusted analyses

Does your paper address CONSORT subitem 12b? *

Copy and paste relevant sections from the manuscript (include quotes in quotation marks "like this" to indicate direct quotes from your manuscript), or elaborate on this item by providing additional information not in the ms, or briefly explain why the item is not applicable/relevant for your study

Yes, "Change in visceral anxiety, catastrophizing and fear of food (calculated as change from baseline to 8 weeks) were explored as possible mediators of GI symptoms and quality of life at 8 weeks using regression analysis with estimates of indirect effects calculated using a percentile bootstrap estimation approach with 5000 samples implemented with the PROCESS macro Version 3.5 [47]. Both direct and indirect effects are reported. The direct effect quantifies the estimated difference in the dependent variable (GI symptoms or quality of life) between two cases that are equal on the mediator but differ by one unit on treatment assignment, i.e., intervention vs waitlist group. The indirect effect quantifies how much two cases, one assigned to immediate treatment, the other to waitlist, are estimated to differ on the dependent variables (GI symptoms or quality of life) as a result of treatments' influence on the mediator, which in turn influences the dependent variable. Two sets of models were fitted, the first tested the mediator variables separately with simple mediator models, the second fitted a parallel mediator model where the three mediators were tested simultaneously. The baseline level of the dependent variable was included as a covariate in all mediation models."

X26) REB/IRB Approval and Ethical Considerations [recommended as subheading under "Methods"] (not a CONSORT item)

X26-i) Comment on ethics committee approval

	1	2	3	4	5	
subitem not at all important	○	○	○	○	○	essential

Does your paper address subitem X26-i?

Copy and paste relevant sections from the manuscript (include quotes in quotation marks "like this" to indicate direct quotes from your manuscript), or elaborate on this item by providing additional information not in the ms, or briefly explain why the item is not applicable/relevant for your study

Yes, "This study was approved by the Institutional Review Board at the University of Pennsylvania. All participants provided electronic consent prior to participation in the study. The de-identified dataset analyzed in the study is available from the corresponding author upon request. This trial was registered at ClinicalTrials.gov as NCT04170686."

x26-ii) Outline informed consent procedures

Outline informed consent procedures e.g., if consent was obtained offline or online (how? Checkbox, etc.?), and what information was provided (see 4a-ii). See [6] for some items to be included in informed consent documents.

	1	2	3	4	5	
subitem not at all important	○	○	○	○	○	essential

Does your paper address subitem X26-ii?

Copy and paste relevant sections from the manuscript (include quotes in quotation marks "like this" to indicate direct quotes from your manuscript), or elaborate on this item by providing additional information not in the ms, or briefly explain why the item is not applicable/relevant for your study

Yes, "All participants provided electronic consent prior to participation in the study.", "After completing the consent and all the baseline measures, participants were randomly allocated by a research coordinator to either immediate treatment or waitlist control using the coin toss function of random.org.", and "Posts and advertisements included a link to a secure University of Pennsylvania Qualtrics study page. On following the link, potential participants would first see the detailed explanation of the research (Consent Form - See Appendix A) and would consent to completing the baseline questionnaires."

X26-iii) Safety and security procedures

Safety and security procedures, incl. privacy considerations, and any steps taken to reduce the likelihood or detection of harm (e.g., education and training, availability of a hotline)

	1	2	3	4	5	
subitem not at all important	○	○	○	○	○	essential

Does your paper address subitem X26-iii?

Copy and paste relevant sections from the manuscript (include quotes in quotation marks "like this" to indicate direct quotes from your manuscript), or elaborate on this item by providing additional information not in the ms, or briefly explain why the item is not applicable/relevant for your study

Yes, "If at any point a participant had indicated a significant increase in depressive symptoms or the onset of suicidal ideation, the team would have alerted the PI (a licensed clinical psychologist) who would have reached out to that individual to conduct a risk assessment and offer referrals to local resources. No such adverse events occurred."

RESULTS

13a) For each group, the numbers of participants who were randomly assigned, received intended treatment, and were analysed for the primary outcome

NPT: The number of care providers or centers performing the intervention in each group and the number of patients treated by each care provider in each center

Does your paper address CONSORT subitem 13a? *

Copy and paste relevant sections from the manuscript (include quotes in quotation marks "like this" to indicate direct quotes from your manuscript), or elaborate on this item by providing additional information not in the ms, or briefly explain why the item is not applicable/relevant for your study

Yes, "See Figure 1 for the Consort Diagram of participant flow through the study."

13b) For each group, losses and exclusions after randomisation, together with reasons

Does your paper address CONSORT subitem 13b? (NOTE: Preferably, this is shown in a CONSORT flow diagram) *

Copy and paste relevant sections from the manuscript (include quotes in quotation marks "like this" to indicate direct quotes from your manuscript), or elaborate on this item by providing additional information not in the ms, or briefly explain why the item is not applicable/relevant for your study

Yes, "See Figure 1 for the Consort Diagram of participant flow through the study."

13b-i) Attrition diagram

Strongly recommended: An attrition diagram (e.g., proportion of participants still logging in or using the intervention/comparator in each group plotted over time, similar to a survival curve) or other figures or tables demonstrating usage/dose/engagement.

	1	2	3	4	5	
subitem not at all important	○	○	○	○	○	essential

Does your paper address subitem 13b-i?

Copy and paste relevant sections from the manuscript or cite the figure number if applicable (include quotes in quotation marks "like this" to indicate direct quotes from your manuscript), or elaborate on this item by providing additional information not in the ms, or briefly explain why the item is not applicable/relevant for your study

Your answer

14a) Dates defining the periods of recruitment and follow-up

Does your paper address CONSORT subitem 14a? *

Copy and paste relevant sections from the manuscript (include quotes in quotation marks "like this" to indicate direct quotes from your manuscript), or elaborate on this item by providing additional information not in the ms, or briefly explain why the item is not applicable/relevant for your study

Yes, "All recruitment and follow-up occurred between 10/01/2019 and 11/01/2020. The trial ended upon successful completion."

14a-i) Indicate if critical “secular events” fell into the study period

Indicate if critical “secular events” fell into the study period, e.g., significant changes in Internet resources available or “changes in computer hardware or Internet delivery resources”

	1	2	3	4	5	
subitem not at all important	○	○	○	○	○	essential

Does your paper address subitem 14a-i?

Copy and paste relevant sections from the manuscript (include quotes in quotation marks "like this" to indicate direct quotes from your manuscript), or elaborate on this item by providing additional information not in the ms, or briefly explain why the item is not applicable/relevant for your study

No critical “secular events” fell into the study period

14b) Why the trial ended or was stopped (early)

Does your paper address CONSORT subitem 14b? *

Copy and paste relevant sections from the manuscript (include quotes in quotation marks "like this" to indicate direct quotes from your manuscript), or elaborate on this item by providing additional information not in the ms, or briefly explain why the item is not applicable/relevant for your study

Yes, "The trial ended upon successful completion."

15) A table showing baseline demographic and clinical characteristics for each group

NPT: When applicable, a description of care providers (case volume, qualification, expertise, etc.) and centers (volume) in each group

Does your paper address CONSORT subitem 15? *

Copy and paste relevant sections from the manuscript (include quotes in quotation marks "like this" to indicate direct quotes from your manuscript), or elaborate on this item by providing additional information not in the ms, or briefly explain why the item is not applicable/relevant for your study

Yes, "See (Table 1) for all means and standard deviations across all assessment timepoints."

15-i) Report demographics associated with digital divide issues

In ehealth trials it is particularly important to report demographics associated with digital divide issues, such as age, education, gender, social-economic status, computer/Internet/ehealth literacy of the participants, if known.

	1	2	3	4	5	
subitem not at all important	○	○	○	○	○	essential

Does your paper address subitem 15-i? *

Copy and paste relevant sections from the manuscript (include quotes in quotation marks "like this" to indicate direct quotes from your manuscript), or elaborate on this item by providing additional information not in the ms, or briefly explain why the item is not applicable/relevant for your study

While we do report age, gender and employment status, we do not have further information on demographics associated with digital divide issues. However, given that this was a purely web-based trial, and that recruitment was done online, all participants were sufficiently literate in eHealth solutions.

16) For each group, number of participants (denominator) included in each analysis and whether the analysis was by original assigned groups

16-i) Report multiple “denominators” and provide definitions

Report multiple “denominators” and provide definitions: Report N’s (and effect sizes) “across a range of study participation [and use] thresholds” [1], e.g., N exposed, N consented, N used more than x times, N used more than y weeks, N participants “used” the intervention/comparator at specific pre-defined time points of interest (in absolute and relative numbers per group). Always clearly define “use” of the intervention.

	1	2	3	4	5	
subitem not at all important	○	○	○	○	○	essential

Does your paper address subitem 16-i? *

Copy and paste relevant sections from the manuscript (include quotes in quotation marks "like this" to indicate direct quotes from your manuscript), or elaborate on this item by providing additional information not in the ms, or briefly explain why the item is not applicable/relevant for your study

Yes, found in: "Table 1: Means and Standard Deviations for all Outcome Measures Across Trial"

16-ii) Primary analysis should be intent-to-treat

Primary analysis should be intent-to-treat, secondary analyses could include comparing only “users”, with the appropriate caveats that this is no longer a randomized sample (see 18-i).

	1	2	3	4	5	
subitem not at all important	○	○	○	○	○	essential

Does your paper address subitem 16-ii?

Copy and paste relevant sections from the manuscript (include quotes in quotation marks "like this" to indicate direct quotes from your manuscript), or elaborate on this item by providing additional information not in the ms, or briefly explain why the item is not applicable/relevant for your study

"Both intent-to-treat and completer analyses at post-treatment revealed significant improvement for the immediate treatment group compared to the waitlist control group on both primary and secondary outcome measures."

17a) For each primary and secondary outcome, results for each group, and the estimated effect size and its precision (such as 95% confidence interval)

Does your paper address CONSORT subitem 17a? *

Copy and paste relevant sections from the manuscript (include quotes in quotation marks "like this" to indicate direct quotes from your manuscript), or elaborate on this item by providing additional information not in the ms, or briefly explain why the item is not applicable/relevant for your study

Yes, see "Table 1: Means and Standard Deviations for all Outcome Measures Across Trial"

17a-i) Presentation of process outcomes such as metrics of use and intensity of use

In addition to primary/secondary (clinical) outcomes, the presentation of process outcomes such as metrics of use and intensity of use (dose, exposure) and their operational definitions is critical. This does not only refer to metrics of attrition (13-b) (often a binary variable), but also to more continuous exposure metrics such as “average session length”. These must be accompanied by a technical description how a metric like a “session” is defined (e.g., timeout after idle time) [1] (report under item 6a).

	1	2	3	4	5	
subitem not at all important	○	○	○	○	○	essential

Does your paper address subitem 17a-i?

Copy and paste relevant sections from the manuscript (include quotes in quotation marks "like this" to indicate direct quotes from your manuscript), or elaborate on this item by providing additional information not in the ms, or briefly explain why the item is not applicable/relevant for your study

Yes, "Because the app itself tracks objective progress through the modules, we were able to examine the effect of “dose” (i.e. modules accessed) on outcome. For the immediate treatment group, dosage was marginally correlated improvement in HRQL ($r = .33$, $P =.072$) and depression ($r = .33$, $P = .075$). This suggests that the more participants used the app, the more their quality of life improved and depressive symptoms improved."

17b) For binary outcomes, presentation of both absolute and relative effect sizes is recommended

Does your paper address CONSORT subitem 17b? *

Copy and paste relevant sections from the manuscript (include quotes in quotation marks "like this" to indicate direct quotes from your manuscript), or elaborate on this item by providing additional information not in the ms, or briefly explain why the item is not applicable/relevant for your study

N/A

18) Results of any other analyses performed, including subgroup analyses and adjusted analyses, distinguishing pre-specified from exploratory

Does your paper address CONSORT subitem 18? *

Copy and paste relevant sections from the manuscript (include quotes in quotation marks "like this" to indicate direct quotes from your manuscript), or elaborate on this item by providing additional information not in the ms, or briefly explain why the item is not applicable/relevant for your study

Yes, "The simple mediator models for GI symptom severity show that change in visceral anxiety, catastrophizing and fear of food are all significant mediators of the relationship between treatment and GI symptom severity. Participants assigned to immediate treatment have a greater decrease in visceral anxiety, catastrophizing and fear of food and participants who have a greater decrease in visceral anxiety (indirect effect =-4.3, BCCI 95% -7.0 to -1.8, P=.002), catastrophizing (indirect effect =-3.7, BCCI 95% -7.1 to -1.2, P=.007) and fear of food (indirect effect =-9.8, BCCI 95% -7.2 to -1.5, P=.003) have lower GI symptom severity at 8 weeks while controlling for baseline GI symptom severity.", "Participants assigned to immediate treatment have a greater decrease in visceral anxiety, catastrophizing and fear of food and participants who have a greater decrease in visceral anxiety (indirect effect =-12.2, BCCI 95% -18.2 to -6.4, P<.001), catastrophizing (indirect effect =-15.4, BCCI 95% -21.6 to -9.6, P<.001) and fear of food (indirect effect =-4.0, BCCI 95% -16.3 to -3.8, P=.008) have lower scores on IBS QoL at 8 weeks while controlling for baseline IBS QoL."

18-i) Subgroup analysis of comparing only users

A subgroup analysis of comparing only users is not uncommon in ehealth trials, but if done, it must be stressed that this is a self-selected sample and no longer an unbiased sample from a randomized trial (see 16-iii).

	1	2	3	4	5	
subitem not at all important	○	○	○	○	○	essential

Does your paper address subitem 18-i?

Copy and paste relevant sections from the manuscript (include quotes in quotation marks "like this" to indicate direct quotes from your manuscript), or elaborate on this item by providing additional information not in the ms, or briefly explain why the item is not applicable/relevant for your study

Yes, "Univariate Analysis of data revealed that Rome IV criteria moderated the effectiveness of the treatment. That is, there was a significant interaction between condition and Rome IV status such that the app was more helpful to the participants who reported meeting stringent Rome IV criteria for IBS at baseline than those who did not for both GI symptoms [F(3,76) = 2.919, P < .05] and HRQL [F(3,76) = 6.652, P = 0.001]. Interestingly, the only difference at baseline between those who met criteria and those who did not was severity of GI symptoms [t(144) = 3.75, P < .001]. No other baseline variables were significantly different. When the sample is restricted to only those individuals who met strict Rome IV criteria, the advantage of the treatment group over the waitlist group is even more marked for improvement in GI symptoms [F(1,56) = 30.2, P < .001]; HRQL [F(1,56) = 47.42, P < .001], catastrophizing [F(1,56) = 51.10, P < .001], visceral anxiety [F(1,56) = 28.84, P < .001] and fear of food [F(1,56) = 22.11, P < .001]."

19) All important harms or unintended effects in each group

(for specific guidance see CONSORT for harms)

Does your paper address CONSORT subitem 19? *

Copy and paste relevant sections from the manuscript (include quotes in quotation marks "like this" to indicate direct quotes from your manuscript), or elaborate on this item by providing additional information not in the ms, or briefly explain why the item is not applicable/relevant for your study

Yes, "No such adverse events occurred."

19-i) Include privacy breaches, technical problems

Include privacy breaches, technical problems. This does not only include physical “harm” to participants, but also incidents such as perceived or real privacy breaches [1], technical problems, and other unexpected/unintended incidents. “Unintended effects” also includes unintended positive effects [2].

	1	2	3	4	5	
subitem not at all important	○	○	○	○	○	essential

Does your paper address subitem 19-i?

Copy and paste relevant sections from the manuscript (include quotes in quotation marks "like this" to indicate direct quotes from your manuscript), or elaborate on this item by providing additional information not in the ms, or briefly explain why the item is not applicable/relevant for your study

No privacy breaches or technical problems occurred

19-ii) Include qualitative feedback from participants or observations from staff/researchers

Include qualitative feedback from participants or observations from staff/researchers, if available, on strengths and shortcomings of the application, especially if they point to unintended/unexpected effects or uses. This includes (if available) reasons for why people did or did not use the application as intended by the developers.

	1	2	3	4	5	
subitem not at all important	○	○	○	○	○	essential

Does your paper address subitem 19-ii?

Copy and paste relevant sections from the manuscript (include quotes in quotation marks "like this" to indicate direct quotes from your manuscript), or elaborate on this item by providing additional information not in the ms, or briefly explain why the item is not applicable/relevant for your study

N/A

DISCUSSION

22) Interpretation consistent with results, balancing benefits and harms, and considering other relevant evidence

NPT: In addition, take into account the choice of the comparator, lack of or partial blinding, and unequal expertise of care providers or centers in each group

22-i) Restate study questions and summarize the answers suggested by the data, starting with primary outcomes and process outcomes (use)

Restate study questions and summarize the answers suggested by the data, starting with primary outcomes and process outcomes (use).

	1	2	3	4	5	
subitem not at all important	○	○	○	○	○	essential

Does your paper address subitem 22-i? *

Copy and paste relevant sections from the manuscript (include quotes in quotation marks "like this" to indicate direct quotes from your manuscript), or elaborate on this item by providing additional information not in the ms, or briefly explain why the item is not applicable/relevant for your study

Yes, "The purpose of this study was two-fold. First, we tested the efficacy of a cognitive-behavioral intervention for IBS delivered via a digital self-help app, with no therapist feedback. Completer analyses yielded statistically significant effects, with treatment having a positive impact on both GI symptom severity and quality of life. Intent-to-treat sensitivity analysis using multiple imputation replicated those findings. After treatment, individuals reported significantly lower levels of IBS symptoms and less impairment to their quality of life. Effects size for the primary outcomes and most of the secondary outcomes were all in the very large range. This eight-week intervention appears to have substantially reduced the burden of illness compared to wait-list controls.

Secondly, we tested whether reductions in IBS specific catastrophic thinking, visceral sensitivity, and fear of food might mediate the efficacy of treatment. Reductions in these three variables did appear to mediate the impact of treatment on health related quality of life, though not on GI symptoms themselves. The app worked by reducing catastrophic thinking, visceral sensitivity to GI symptoms, and fear of food, which in turn improved individuals' quality of life. This is consistent with prior findings about the impact of CBT on IBS."

22-ii) Highlight unanswered new questions, suggest future research

Highlight unanswered new questions, suggest future research.

	1	2	3	4	5	
subitem not at all important	○	○	○	○	○	essential

Does your paper address subitem 22-ii?

Copy and paste relevant sections from the manuscript (include quotes in quotation marks "like this" to indicate direct quotes from your manuscript), or elaborate on this item by providing additional information not in the ms, or briefly explain why the item is not applicable/relevant for your study

Yes, "Moving forward, it may be important for the app to encourage people who do not meet Rome IV criteria to consult with their physicians about other possible causes of their symptoms.", "In addition, people did not drop out entirely at random. Participants who dropped out during the initial treatment phase had significantly higher rates of visceral anxiety and fear of food at baseline (although there were no other significant differences). Since CBT for IBS typically encourages acceptance of visceral sensations, and reduction of behavioral avoidance (especially avoidance of food and food related social situations), the treatment may have seemed particularly challenging for those individuals. Those might be the folks who need more personal guidance, encouragement and support from in person therapy.", "A third limitation of the study was the inability to statistically establish the temporal precedence of the proposed mediators of change. In the study design, there was no mid-point survey to show that visceral anxiety, catastrophizing, and fear of food changed before quality of life improved. We did not include this intermediate survey during the treatment phase because we were concerned that it would increase attrition of participants, though a future study of the app would benefit from data obtained at this point." "Although the PHQ has been used in many other clinical trials of behavioral health interventions, and it did show significant improvement over the course of this trial in the completer sample (but not the intent-to-treat analyses), we were dissatisfied with its sensitivity to treatment effects. Future studies of the app will employ more sensitive measures (such as the Beck Depression Inventory)."

20) Trial limitations, addressing sources of potential bias, imprecision, and, if relevant, multiplicity of analyses

20-i) Typical limitations in ehealth trials

Typical limitations in ehealth trials: Participants in ehealth trials are rarely blinded. Ehealth trials often look at a multiplicity of outcomes, increasing risk for a Type I error. Discuss biases due to non-use of the intervention/usability issues, biases through informed consent procedures, unexpected events.

	1	2	3	4	5	
subitem not at all important	O	O	O	O	O	essential

Does your paper address subitem 20-i? *

Copy and paste relevant sections from the manuscript (include quotes in quotation marks "like this" to indicate direct quotes from your manuscript), or elaborate on this item by providing additional information not in the ms, or briefly explain why the item is not applicable/relevant for your study

Yes, "Finally, the last phase of the trial occurred during the COVID-19 global pandemic. Since all waitlist participants had already been crossed over to the active treatment phase, the 3 month follow-up data may reflect less the enduring effects of the treatment and more the massive social, economic and personal upheaval the pandemic has caused. Indeed, the end of the treatment phase for all subjects coincided with the COVID-19 pandemic's arrival in the United States. With massive shutdowns and quarantines, it is highly likely that distress increased for all participants. The fact that treatment gains were generally maintained and that participants remained much improved over baseline even in the face of the pandemic, is encouraging."

21) Generalisability (external validity, applicability) of the trial findings

NPT: External validity of the trial findings according to the intervention, comparators, patients, and care providers or centers involved in the trial

21-i) Generalizability to other populations

Generalizability to other populations: In particular, discuss generalizability to a general Internet population, outside of a RCT setting, and general patient population, including applicability of the study results for other organizations

	1	2	3	4	5	
subitem not at all important	○	○	○	○	○	essential

Does your paper address subitem 21-i?

Copy and paste relevant sections from the manuscript (include quotes in quotation marks "like this" to indicate direct quotes from your manuscript), or elaborate on this item by providing additional information not in the ms, or briefly explain why the item is not applicable/relevant for your study

Yes, "The intervention was not restricted by geography or scheduling constraints, and required no face-to-face contact with a clinician, aspects which dramatically increase the accessibility and portability of treatment. Despite its relatively benign physical profile, IBS can be an extraordinarily debilitating condition. Finding novel ways to disseminate evidence based, effective treatments remains an important challenge, and Zemedy is a promising and effective way to help those suffering from IBS."

21-ii) Discuss if there were elements in the RCT that would be different in a routine application setting

Discuss if there were elements in the RCT that would be different in a routine application setting (e.g., prompts/reminders, more human involvement, training sessions or other co-interventions) and what impact the omission of these elements could have on use, adoption, or outcomes if the intervention is applied outside of a RCT setting.

	1	2	3	4	5	
subitem not at all important	○	○	○	○	○	essential

Does your paper address subitem 21-ii?

Copy and paste relevant sections from the manuscript (include quotes in quotation marks "like this" to indicate direct quotes from your manuscript), or elaborate on this item by providing additional information not in the ms, or briefly explain why the item is not applicable/relevant for your study

Yes, "Participants who dropped out during the initial treatment phase had significantly higher rates of visceral anxiety and fear of food at baseline (although there were no other significant differences). Since CBT for IBS typically encourages acceptance of visceral sensations, and reduction of behavioral avoidance (especially avoidance of food and food related social situations), the treatment may have seemed particularly challenging for those individuals. Those might be the folks who need more personal guidance, encouragement and support from in person therapy."

OTHER INFORMATION

23) Registration number and name of trial registry

Does your paper address CONSORT subitem 23? *

Copy and paste relevant sections from the manuscript (include quotes in quotation marks "like this" to indicate direct quotes from your manuscript), or elaborate on this item by providing additional information not in the ms, or briefly explain why the item is not applicable/relevant for your study

Yes, "This trial was registered at ClinicalTrials.gov as NCT04170686."

24) Where the full trial protocol can be accessed, if available

Does your paper address CONSORT subitem 24? *

Cite a Multimedia Appendix, other reference, or copy and paste relevant sections from the manuscript (include quotes in quotation marks "like this" to indicate direct quotes from your manuscript), or elaborate on this item by providing additional information not in the ms, or briefly explain why the item is not applicable/relevant for your study

https://clinicaltrials.gov/ct2/show/NCT04170686

25) Sources of funding and other support (such as supply of drugs), role of funders

Does your paper address CONSORT subitem 25? *

Copy and paste relevant sections from the manuscript (include quotes in quotation marks "like this" to indicate direct quotes from your manuscript), or elaborate on this item by providing additional information not in the ms, or briefly explain why the item is not applicable/relevant for your study

Yes, "Bold Health provided some funding to pay for recruitment advertisements and participant incentives."

X27) Conflicts of Interest (not a CONSORT item)

X27-i) State the relation of the study team towards the system being evaluated

In addition to the usual declaration of interests (financial or otherwise), also state the relation of the study team towards the system being evaluated, i.e., state if the authors/evaluators are distinct from or identical with the developers/sponsors of the intervention.

	1	2	3	4	5	
subitem not at all important	○	○	○	○	○	essential

Does your paper address subitem X27-i?

Copy and paste relevant sections from the manuscript (include quotes in quotation marks "like this" to indicate direct quotes from your manuscript), or elaborate on this item by providing additional information not in the ms, or briefly explain why the item is not applicable/relevant for your study

Yes, "Dr. Onwude has a financial ownership stake in Bold Health which developed and markets the Zemedy App. Dr. Hunt, Ms. Miguez, Mr. Dukas and Dr. White have no FCOI to declare."

About the CONSORT EHEALTH checklist

As a result of using this checklist, did you make changes in your manuscript? *

- (●) yes, major changes
- ( ) yes, minor changes
- ( ) no

What were the most important changes you made as a result of using this checklist?

Expanded on the content of subsections of the paper

How much time did you spend on going through the checklist INCLUDING making changes in your manuscript *

5 days

As a result of using this checklist, do you think your manuscript has improved? *

- (●) yes
- ( ) no
- ( ) Other:

Would you like to become involved in the CONSORT EHEALTH group?

This would involve for example becoming involved in participating in a workshop and writing an "Explanation and Elaboration" document

- (x) yes
- ( ) no
- ( ) Other:

Clear selection

Any other comments or questions on CONSORT EHEALTH

Your answer

STOP - Save this form as PDF before you click submit

To generate a record that you filled in this form, we recommend to generate a PDF of this page (on a Mac, simply select "print" and then select "print as PDF") before you submit it.

When you submit your (revised) paper to JMIR, please upload the PDF as supplementary file.

Don't worry if some text in the textboxes is cut off, as we still have the complete information in our database. Thank you!

Final step: Click submit !

Click submit so we have your answers in our database!

Submit

Never submit passwords through Google Forms.

This content is neither created nor endorsed by Google. Report Abuse - Terms of Service - Privacy Policy

Google Forms
